# Linking within- and between-host scales for understanding the evolutionary dynamics of quantitative antimicrobial resistance

**DOI:** 10.1007/s00285-023-02008-1

**Published:** 2023-10-27

**Authors:** Martin L. Mann-Manyombe, Abdoulaye Mendy, Ousmane Seydi, Ramsès Djidjou-Demasse

**Affiliations:** 1 MIVEGEC, Université de Montpellier, CNRS, IRD, Montpellier, France; 2 Département Tronc Commun, École Polytechnique de Thiès, Thies, Senegal

**Keywords:** Antimicrobial resistance, Evolutionary dynamics, Nested models, Non-linear dynamical system, 37N25, 35BXX, 92D30

## Abstract

Understanding both the epidemiological and evolutionary dynamics of antimicrobial resistance is a major public health concern. In this paper, we propose a nested model, explicitly linking the within- and between-host scales, in which the level of resistance of the bacterial population is viewed as a continuous quantitative trait. The within-host dynamics is based on integro-differential equations structured by the resistance level, while the between-host scale is additionally structured by the time since infection. This model simultaneously captures the dynamics of the bacteria population, the evolutionary transient dynamics which lead to the emergence of resistance, and the epidemic dynamics of the host population. Moreover, we precisely analyze the model proposed by particularly performing the uniform persistence and global asymptotic results. Finally, we discuss the impact of the treatment rate of the host population in controlling both the epidemic outbreak and the average level of resistance, either if the within-host scale therapy is a success or failure. We also explore how transitions between infected populations (treated and untreated) can impact the average level of resistance, particularly in a scenario where the treatment is successful at the within-host scale.

## Introduction

Antimicrobial resistance (AMR) is one of the major challenges we face in the modern area (Larsson and Flach 2022). An antimicrobial substance is a chemical agent interacting with the physiology of a bacterial cell. The antimicrobial activity on a given bacterium’s (${\mathcal {S}}$) is an increasing function of its concentration in the medium (${\mathcal {C}}$), such that ${\mathcal {S}}\left( 0\right) =0$ and ${\mathcal {S}}\left( {\mathcal {C}}\right) \rightarrow {\mathcal {S}}_{\mathrm{{sat}}}$ as ${\mathcal {C}}\rightarrow {\mathcal {C}}_{\mathrm{{sat}}}$, where ${\mathcal {S}}_{\mathrm{{sat}}}$ and ${\mathcal {C}}_{\mathrm{{sat}}}$ are saturating constants. This intuitive approach implies that there exists ${\mathcal {C}}^{\star }$ in $\left( 0,{\mathcal {C}}_{\mathrm{{sat}}}\right)$ such that ${\mathcal {S}}\left( {\mathcal {C}}^{\star }\right)$ is equal to the intrinsic rate of increase and reverses the growth of a bacterial population. Such a threshold concentration at which a bacterial population does not grow (at least in *in vitro*) is called the Minimum Inhibitory Concentration (MIC). The level of resistance to a given antimicrobial is then a continuous trait by nature referred to as antimicrobial quantitative resistance (qAMR), at least at the population level, and qAMR is key to better understanding the evolutionary dynamics of AMR (Djidjou-Demasse et al. 2023). Here, we introduce a quantitative descriptor $x \in {\mathbb {R}}$—a label of the bacterial strain with resistance level *x*– describing the level of resistance. Most of the modelling approaches devoted to AMR tackling the case of qualitative (or “binary”) resistance are generally based on the dynamical interaction between two parasite strains resulting in a discrete and finite formulation of MICs (Blanquart 2019). This analysis ignores the evolutionary short-term transient dynamics which lead to the emergence of resistance (e.g., Lipsitch and Levin 1997; Kepler and Perelson 1998; Day and Read 2016; Djidjou-Demasse et al. 2021; Tazzyman and Bonhoeffer 2014; Millan et al. 2014; D’Agata et al. 2008).

Here, we proposed a nested (or embedded) model explicitly linking the within- and between-host evolutionary dynamics. Such a nested structure is particularly important because, over the past few decades, it is clear that ecological and evolutionary dynamics are influenced by processes operating across scales (Elderd et al. 2022). Very few studies considered the continuous nature of AMR in the context of this work (e.g., Djidjou-Demasse et al. 2023), and few studies have implemented a nested model in this context so far (e.g., Beardmore et al. 2017; Shen et al. 2019). The bacterial population is assumed to be phenotypically (and genetically) diverse through the level of antimicrobial resistance *x*. This quantitative trait affects different components of the bacterial population life cycle, such as growth and death rates. In addition to those effects on the death and birth rates, bacterial population resistance level also mitigates the antimicrobial efficiency with respect to that population. From a theoretical point of view, properties of the within-host model proposed here are based on previous analytical quantitative genetics results developed in Djidjou-Demasse et al. (2017) and Burie et al. (2020).

An integro-differential equation is used to model the within-host dynamics of the bacterial population. Such a within model formulation is previously proposed in Djidjou-Demasse et al. (2023). Each host individual is classified as either a treated host, labeled as *T*, or an untreated host, labeled as *U*. The model tracks the dynamics of a bacterial population within a treated host ($b^T$) or an untreated host ($b^U$). At time $\tau$, the bacteria density with resistance level $y\in {\mathbb {R}}$ within a treated and untreated host is quantified by $b_i^T(\tau ,y)$ and $b_i^U(\tau ,y)$ respectively. The subscript “*i*” (with $i\in {\mathcal {I}}=\{1,2,\cdots ,n\}$) represents an individual immune system and then allows taking into account the immune system heterogeneity in the host population. A bacteria with resistance level *y* generate offspring with resistance level *x* at a per-capita rate $J(x-y) p(y) b_i^{\vartheta }(\tau ,y)$, where *p*(*y*) is the bacterial intrinsic growth rate, and $J(x-y)$ is the probability for a bacterial population with resistance level *y* to mutate towards a level *x* during the reproduction process. Therefore, the total number of bacteria produced at time $\tau$ with a resistance level *x* is quantified by $\left( 1+\int _{{\mathbb {R}}} b_i^{\vartheta }(\tau ,x)dx\right) ^{-\kappa } \int _{{\mathbb {R}}}J(x-y)p(y) b_i^{\vartheta }(\tau ,y) \textrm{d}y$, where $\kappa$ is a positive parameter. The parameter $\kappa >0$ is introduced to impose the bacterial population homeostasis. The within-host model reads as

1.1 $$\begin{aligned} {\left\{ \begin{array}{ll} \displaystyle \partial _{\tau }b_i^{\vartheta }(\tau ,x)= \frac{1}{\left( 1+B_i^{\vartheta }(\tau )\right) ^{\kappa }} \int _{{\mathbb {R}}}J(x-y)p(y)b_i^{\vartheta }(\tau ,y)\textrm{d}y - \xi _i^{\vartheta }(x)b_i^{\vartheta }(\tau ,x),\\ \displaystyle b_i^{\vartheta }(0,\cdot )=b_{0}(\cdot ), \end{array}\right. } \end{aligned}$$

where $B_i^{\vartheta }(\tau )= \int _{{\mathbb {R}}} b_i^{\vartheta }(\tau ,x)\textrm{d}x,$ is the total bacteria load. The term $\xi _i^{\vartheta }$ accounts for the individual clearance of bacterial cells with resistance level *x*, either by the immune system ($\mu _i$) or by the efficiency of antimicrobial pressure (*k*). Thus, $\xi _i^U(x)= \mu _i(x)$, for untreated host, and $\xi _i^T(x)=\mu _i(x)+k(x)$, for treated host. Here, it is assumed that bacteria are subject to a biocidal antimicrobial pressure, .ie. killing and not diminishing the birth rate of bacteria. Note that, the within-host model (1.1) allows to follow evolutionary parameters such as the average level of resistance for treated ($\bar{x}_i^T(\tau )$) and untreated ($\bar{x}_i^U(\tau )$) individuals $\tau$-time post infection given by

$$\begin{aligned} {\bar{x}}_i^\vartheta (\tau )= \int _{{\mathbb {R}}}x\frac{b_i^\vartheta (\tau ,x)}{B_i^\vartheta (\tau )}\textrm{d}x, \quad \vartheta \in \{T,U\}. \end{aligned}$$

At the between-host scale, the host population is subdivided into three states. At any time *t*, an individual—with the immune system’s response level $i \in {\mathcal {I}}$—can be susceptible to the infection $S_i(t)$ or infected $I_i^\vartheta (t,\tau ,\bar{x}_i^{\vartheta }(\tau ))$, $\vartheta =\{T,U\}$. The variables $\tau$ and $\bar{x}_i^{\vartheta }(\tau )$ respectively represent the time post-infection and the average resistance level of the infected host. It is important to clearly understand the meaning of infected individuals $I^\vartheta (t,\tau ,{\bar{x}}_i^\vartheta (\tau ))$. Indeed, each infected individuals is potentially infected with multiple bacteria strains with variable frequencies and resistance levels. Therefore, $\bar{x}_i^\vartheta (\tau )$ represent the individual resistance level quantified by the within-host dynamics through the above formula. However, for simplicity, and without loss of generality, we will note $I_i^\vartheta (t,\tau ,{\bar{x}}_i^\vartheta (\tau ))\equiv I_i^\vartheta (t,\tau )$ for $\vartheta \in \{T,U\}$ and $i\in {\mathcal {I}}$.

Individual transmission and loss rates at the between-host scale, $\tau$-time since infection, $\beta _i^{\vartheta }(\tau )$ and $\alpha _i^{\vartheta }(\tau )$, are linked to the within-host dynamics at time $\tau$. As an example, these parameters can be represented as Holling functions of type II (or similarly the Beddington-DeAngelis functional response) such that, for all $\vartheta \in \{T,U\}$,

1.2 $$\begin{aligned} \beta _i^{\vartheta }(\tau )= \frac{\beta _0B_i^{\vartheta }(\tau )}{r_0+ B_i^{\vartheta }(\tau )}\quad \text{ and }\quad \alpha _i^{\vartheta }(\tau )=\frac{\alpha _0B_i^{\vartheta }(\tau )}{r_0+ B_i^{\vartheta }(\tau )}+\gamma ^{\vartheta }(\tau ), \end{aligned}$$

where $\beta _0$ and $\alpha _0$ are scaling constants, and $r_0$ is the half-saturation constant for the total bacterial load $B_i^{\vartheta }$. Note that, for the loss rate of infected individuals $\alpha _i^{\vartheta }$, the term $\alpha _0B_i^{\vartheta }/(r_0+ B_i^{\vartheta })$ represents the loss due to the disease induced mortality while $\gamma ^{\vartheta }$ is the loss due to recovery. We can assume that the function $\gamma ^{\vartheta }$ is of the form

1.3 $$\begin{aligned} \gamma _{i}^\vartheta (\tau )= \left\{ \begin{array}{ll} 0, &{} \hbox {if} \quad B_i^\vartheta (\tau )> B_{min}, \\ 1, &{} \hbox {if}\quad B_i^\vartheta (\tau )\le B_{min}, \end{array} \right. \end{aligned}$$

where $B_{min}$ is the threshold below which the infection becomes undetectable such that the infected individuals is considered as recovered.

The force of infection induced by infected individuals at time *t* is then given by

$$\begin{aligned} \lambda (t)=\sum _{i \in {\mathcal {I}}} \int _0^{\infty }[\beta _i^T(\tau ) I_i^T(t,\tau ) + \beta _i^U(\tau )I_i^U(t,\tau )]\textrm{d}\tau . \end{aligned}$$

The nested model proposed here then makes it possible to simultaneously track the epidemiological dynamics of the host population as well as evolutionary quantities such as the average level of resistance at both the within- and between-host scales. Such an approach is original and to our knowledge, no study has considered nested models for the evolutionary dynamics of AMR, viewed as a continuous quantitative trait.

The dynamics of newly infected individuals (i.e. $\tau =0$) in each group (treated or untreated) is thus defined by (for $\vartheta \in \{T,U\}$)

1.4 $$\begin{aligned} I_i^\vartheta (t,\tau =0)=q_i^\vartheta \lambda (t)S_i(t), \end{aligned}$$

where $q_i^T\in (0,1)$ is the treatment rate in the host population and $q_i^U=1-q_i^T$. During their infection, treated individuals can stop the treatment at rate $\omega _U^T(\tau )$, and untreated infections can join the treated group at rate $\omega _T^U(\tau )$. The loss rate of infected individuals $\tau$-time post infection occurs at rate $\alpha _i^{\vartheta }(\tau )$. Susceptible individuals are recruited at a constant rate $\Lambda _i$ and the natural death rate of the host population is $\mu _h$. The between-host model then reads

1.5 $$\begin{aligned} {\left\{ \begin{array}{ll} \displaystyle \dot{S}_i(t)=\Lambda _i-S_i(t) \lambda (t) -\mu _h S_i(t),\\ \displaystyle \left( \partial _t+\partial _{\tau }\right) I_i^T(t,\tau )= - \left( \alpha _i^T(\tau ) +\omega _U^T(\tau ) +\mu _h\right) I_i^T(t,\tau ) +\omega _T^U(\tau )I_i^U(t,\tau ), \\ \displaystyle \left( \partial _t+\partial _{\tau }\right) I_i^U(t,\tau )= - \left( \alpha _i^U(\tau ) +\omega _T^U(\tau )+\mu _h\right) I_i^U(t,\tau )+\omega _U^T(\tau )I_i^T(t,\tau ),\\ S_i(0)=S_{i,0},\quad I_i^{\vartheta }(0,\tau )=I_{i,0}^{\vartheta }(\tau ). \end{array}\right. }\nonumber \\ \end{aligned}$$

**Fig. 1:**
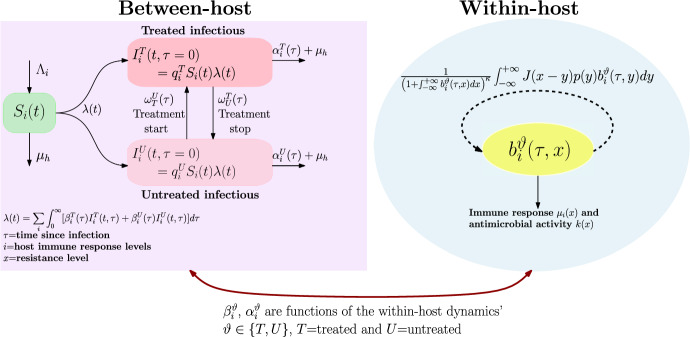
Flow diagram of the nested model. *Within-host model:* the number of bacteria produced at time $$\tau $$ with resistance level *x* is $$\frac{1}{\left( 1+\int _{-\infty }^{+\infty } b_i^{\vartheta }(\tau ,x)\textrm{d}x\right) ^{\kappa }} \int _{-\infty }^{+\infty }J(x-y)p(y)b_i^{\vartheta }(\tau ,y)\textrm{d} y$$, where $$J(x-y)$$ is the probability for a bacterial with resistance level $$y\in {\mathbb {R}}$$ to mutate towards a level $$x\in {\mathbb {R}}$$ and *p*(*y*) is the bacterial intrinsic growth rate. Bacterial cells with resistance level *x*, within an individual with immune system level *i* are cleared either by the immune system at rate $$\mu _i(x)$$ or by the antimicrobial efficiency at rate *k*(*x*). *Between-host model:* susceptible individuals are recruited at a constant rate $$\Lambda _i$$. $$I_i^T(t,\tau )$$ and $$I_i^U(t,\tau )$$ are respectively treated and untreated infected individuals at time *t*, which are infected since time $$\tau $$. The force of infection in the whole population at time *t* is $$ \lambda (t)= \sum _{i \in {\mathcal {I}}} \int _0^{\infty }[\beta _i^T(\tau ) I_i^T(t,\tau ) + \beta _i^U(\tau )I_i^U(t,\tau )]\textrm{d}\tau $$, with $$\beta _i^{\vartheta }(\tau )$$ the disease transmission rate of an infected individual $$\tau $$-time post infection. At the time *t*, new infections occur at rate $$\lambda (t) S_i(t)$$, and are either treated with a probability $$q_i^T$$ or untreated with a probability $$ q_i^U=1-q_i^T$$. The natural death rate of individuals is $$\mu _h$$. If infected since time $$\tau $$, the loss rate is $$\alpha _i^{\vartheta }(\tau )$$. Untreated individuals, and infected since time $$\tau $$ start the treatment at rate $$\omega _T^U(\tau )$$ while treated individuals stop the treatment at rate $$\omega _U^T(\tau )$$

Finally, the nested within-host (1.1) and between-host model (1.4)–(1.5) are summarised by Fig. 1. The main variables and parameters are listed in Table 1. We emphasize that the dynamical properties (that we will recall later) of within-host model (1.1) are precisely analyzed in Djidjou-Demasse et al. (2023). Therefore, our main objective here is devoted to the analysis of the nested model (1.1)–(1.5).

The rest of this work is organized as follows. In Sect. 2, we state the main results of the nested model that are obtained in this work. These include the existence of the globally defined non-negative semiflow and the existence of the unique positive equilibrium for the within-host model, and global threshold analysis results for the between-host model. The model’s typical dynamics are provided in Sect. 3. This includes the within- and between-host models parameterization and the characterization of the evolutionary parameters such as the average levels of resistance. In Sect. 4, we delve into the effects of various parameters on the equilibrium structure of the host population, along with addressing the parameterization issue within nested models. Section 5 focuses on providing preliminary results. Specifically, it addresses the existence and uniqueness of solutions, derivation of the basic reproduction number, and the existence of a unique endemic equilibrium for System (1.4)–(1.5). Finally, Sect. 6 is devoted to the proof of the global asymptotic results.

**Table 1:** Within- and between-host model variables and parameters

Notations	
*t*	Time
$\tau$	Time since infection (infection age)
*x*	Resistance level
*i*	host immune response level
*Model state variables*	
Within-host	
$b_i^T(\tau ,x)$	Bacteria density with resistance level *x* at time $\tau$ in a treated host
$b_i^U(\tau ,x)$	Bacteria density with resistance level *x* at time $\tau$ in an untreated host
Between-host	
$S_i(t)$	Susceptible humans at time *t*
$I_i^T(t,\tau )$	Treated infected humans, infected since time $\tau$
$I_i^U(t,\tau )$	Untreated infected humans, infected since time $\tau$
*Model parameters*	
Within-host	
$\kappa$	Limitation on bacterial growth factor
$J(x-y)$	Mutation probability from resistance level *x* to *y* per cell division
*p*(*x*)	Intrinsic growth rate of bacterial population with resistance level *x*
*k*(*x*)	Killing rate of bacterial population with resistance level *x* due to drug
$\mu _i(x)$	Individual clearance rate of the bacterial cell with resistance level *x* due to the immune response
$\xi _i^{U}(x)=\mu _i(x)$	Bacteria clearance rate with resistance level *x* in untreated hosts
$\xi _i^{T}(x)=k(x)+\mu _i(x)$	Bacteria clearance rate with resistance level *x* in treated hosts due to the immune response and drug
Between-host	
$\beta _i^{\vartheta }(\tau )$, $\vartheta \in \{T,U\}$	Transmission rate of infected individuals
$\alpha _i^{\vartheta }(\tau )$, $\vartheta \in \{T,U\}$	Loss rate of infected individuals
$\omega _U^T(\tau )$	Rate of treatment cessation
$\omega ^U_T(\tau )$	Rate of treatment start
$q_i^T$	Proportion of treated hosts
$q_i^U=1-q_i^T$	Proportion of untreated hosts
$\Lambda _i$	Recruitment rate of susceptibles
$\mu _h$	Human natural death rate

## Main results

This section is devoted to the main results of the nested model (1.1)–(1.5). Such results include the existence of the unique maximal bounded semiflow, and a precise description of the unique positive equilibrium of Model (1.4)–(1.5). By providing global stability results, we will also conduct a precise threshold analysis of the between-host model (1.4)–(1.5).

First of all, for biological feasibility of the nested model (1.1)–(1.5), we make use of the following assumptions. More precisely, the within-host model (1.1) is formulated based on the following assumption

### Assumption 2.1

1. Functions $\mu _i$, *k*, $\xi _i^{\vartheta }$, and *p* are always positive on ${\mathbb {R}}$, with $\vartheta \in \{T,U\}$. Furthermore, *p* is a bounded function on ${\mathbb {R}}$ and $\kappa >0$. Finally, the function $\frac{p}{\xi _i^{\vartheta }}$ is continuous on ${\mathbb {R}}$ and satisfies $\frac{p}{\xi _i^{\vartheta }} > 0$ and $\lim \nolimits _{|x|\rightarrow \infty }\frac{ p}{\xi _i^{\vartheta }}(x)=0$.
2. The mutation kernel *J* is bounded and integrable on ${\mathbb {R}}^+$, positive almost everywhere, and satisfies $\int _{{\mathbb {R}}^+}J(x)dx>0$, $J(-x)=J(x)$, for all *x*.
3. The mutation kernel *J* decays rather rapidly towards infinity in the sense that $J(x)=O\left( \frac{1}{|x|^{\infty }}\right)$ as $|x|\rightarrow \infty$. In other words, $\lim \nolimits _{|x|\rightarrow \infty }|x|^nJ(x)=0$, for all $n\in {\mathbb {N}}$.

Furthermore, the between-host model’s parameters satisfy the following assumption.

### Assumption 2.2

1. Recruitment rate $\Lambda _i$ ($i\in {\mathcal {I}}$) and natural death rate $\mu _h$ are positive constants.
2. The treatment rates $q_i^{\vartheta }$ ($i\in {\mathcal {I}}$, $\vartheta \in \{T,U\}$) are positive constants.
3. The rates $\omega _U^T$, $\omega ^U_T$ belongs to $L^{\infty }({\mathbb {R}}_+)$, with respective essential upper bounds ${\overline{\omega }}_U^T$, ${\overline{\omega }}^U_T$ and positive essential lower bounds $\underline{\omega }_U^T$, $\underline{\omega }^U_T$.
4. Parameters $\beta _i^\vartheta$ and $\alpha _i^\vartheta$ ($i\in {\mathcal {I}}$, $\vartheta \in \{T,U\}$) are such that $\beta _i^\vartheta , \alpha _i^\vartheta \in L^\infty ({\mathbb {R}}_+)$.
5. The transmission rates $\beta _i^\vartheta (\cdot )$,s are Lipschitz continuous almost everywhere on ${\mathbb {R}}_+$.

### Summary key findings on the within-host dynamics

The dynamical properties of the within-host model (1.1) have been precisely investigated in Djidjou-Demasse et al. (2023). The first result of Model (1.1) is about the existence of the unique maximal bounded semiflow. Such a result reads as,

#### Theorem 2.3

Let Assumption 2.1 be satisfied. Let $b_{i0}^{\vartheta }\in L^1_+$. Then,

1. There exists a unique global solution $v(\cdot ,b_{i0}^{\vartheta }):[0,\infty )\rightarrow L_+^1({\mathbb {R}})$ of (1.1) with $v(0,b_{i0}^{\vartheta })=b_{i0}^{\vartheta }$ and $v(\tau ,b_{i0}^{\vartheta })=b_i^{\vartheta }(\tau ,\cdot )$ for all $\tau >0$.
2. The semi-flow defined by $\{v(\tau ,b_{i0}^{\vartheta })\}_{\tau }$ is bounded dissipative and asymptotically smooth, and hence, its admits a global attractor in $L_+^1({\mathbb {R}})$.
3. The semi-flow $\{v(\tau ,b_{i0}^{\vartheta })\}_{\tau }$ is such that for any $b_{i0}^{\vartheta }\in L^1_+({\mathbb {R}})\setminus \{0\}$, $b_i^{\vartheta }(\tau ,x)>0$, for all $\tau >0$, $x\in {\mathbb {R}}$.

The basic reproduction number ${\mathcal {N}}_{i0}^{\vartheta }$—defined as the expected number of bacteria arising from one bacterium in a bacteria-free environment—of the bacteria population with resistance level *x*, within an individual with immune system level *i*, is calculated as

2.1 $$\begin{aligned} {\mathcal {N}}_{i0}^{\vartheta }(x)=\frac{p(x)}{\xi _i^{\vartheta }(x)}, \text { for } \vartheta \in \{U,T\}. \end{aligned}$$

Next, a non-trivial equilibrium of Model (1.1) is strongly related to the principal eigenpair of the below linear integral operator $H^{\vartheta }_{i}$ defined on $L^p({\mathbb {R}})$ (for any $p\ge 1$), by

2.2 $$\begin{aligned} H^{\vartheta }_{i}[v_i^{\vartheta }](x)= \sqrt{{\mathcal {N}}_{i0}^{\vartheta }(x)} \int _{{\mathbb {R}}} J(x-y)\sqrt{{\mathcal {N}}_{i0}^{\vartheta }(y)}\,v_i^{\vartheta }(y)\textrm{d}y. \end{aligned}$$

We then have the following result.

#### Theorem 2.4

Let $r(H^{\vartheta }_{i})$, the spectral radius of the operator $H^{\vartheta }_{i}$, and $\phi >0$ the associated eigenfunction normalized such that $||\phi ||_{L^1}=1$.

1. When $r(H^{\vartheta }_{i})\le 1$, the bacteria-free equilibrium $F_{i0}^{\vartheta }$ is the unique equilibrium of Model (1.1).
2. When $r(H^{\vartheta }_{i})> 1$, in addition to $F_{i0}^{\vartheta }$, Model (1.1) has a unique equilibrium ${\overline{F}}_{i}^{\vartheta }>0$ such that
  2.3 $$\begin{aligned} {\overline{F}}_{i}^{\vartheta }(x)= \left( \frac{\left( r(H^{\vartheta }_{i})\right) ^{\frac{1}{\kappa }}-1}{\int _{{\mathbb {R}}}\frac{\phi }{\sqrt{p\; \xi _i^{\vartheta }}}\textrm{d}y} \right) \frac{\phi (x)}{\sqrt{p(x)\xi _i^{\vartheta }(x)}}. \end{aligned}$$
  Furthermore, the semi-flow $\{v(\tau ,b_{i0}^{\vartheta })\}_{\tau }$ is uniformly persistent, that is, there exists a constant $\eta$ such that for any $b_{i0}^{\vartheta }\in L^1_+({\mathbb {R}}){\setminus }\{0\}$, the unique solution $v(\tau ,b_{i0}^{\vartheta })=b_i^{\vartheta }(\tau ,\cdot )$ of Model (1.1) with initial data $b_{i0}^{\vartheta }$ satisfies $\lim \limits _{\tau \rightarrow \infty }\inf \Vert b_i^{\vartheta }(\tau ,\cdot )\Vert _{L^1}>\eta .$
3. The bacteria-free equilibrium $F^{\vartheta }_{i0}$ of Model (1.1) is asymptotically stable if $r(H^{\vartheta }_{i})<1$ and unstable if $r(H^{\vartheta }_{i})>1$.
4. When $r(H^{\vartheta }_{i})<1$, the bacteria-free equilibrium $F^{\vartheta }_{i0}$ is globally asymptotically stable in $L^1_+({\mathbb {R}})$, that is, for any solution $b_i^{\vartheta }(\tau ,\cdot )$ with initial $b_{i0}^{\vartheta }\in L^1_+({\mathbb {R}}){\setminus }\{0\}$, we have $b_i^{\vartheta }(\tau ,\cdot )\rightarrow 0$ in $L^1_+({\mathbb {R}})$, as $\tau \rightarrow \infty$.

We recall that the within-host model (1.1) is precisely analyzed in Djidjou-Demasse et al. (2023). We then refer to Appendices F–I in Djidjou-Demasse et al. (2023) for the detailed proof of Theorems 2.3 and 2.4. Note that the linear operator $H^{\vartheta }_{i}$ naturally emerges when characterizing the positive equilibrium of the within-host model (1.1) (Djidjou-Demasse et al. 2023).

Furthermore, the estimate (2.3) gives that the endemic equilibrium ${\overline{F}}_{i}^{\vartheta }$ of the within-host model (1.1) basically relied to the principal eigenfunction of the linear operator $H^{\vartheta }_{i}$ for any given probability kernel *J* satisfying Assumption 2.1. However, the profile of the endemic equilibrium ${\overline{F}}_{i}^{\vartheta }$ with respect to $x\in {\mathbb {R}}$ can be precisely described when the mutation kernel *J* depends on a small positive parameter (let say $\varepsilon<\!\!<1$) with the scaling form

2.4 $$\begin{aligned} J_\varepsilon (x)=\varepsilon ^{-1}J\left( \varepsilon ^{-1} x\right) , \end{aligned}$$

where $\varepsilon >0$ represents the mutation variance in the phenotypic space. More precisely, when $\varepsilon >0$ is small, then the endemic equilibrium ${\overline{F}}_{i}^{\vartheta }$ concentrates on the set ${\mathcal {S}}_i^{\vartheta }$ defined by

$$\begin{aligned} {\mathcal {S}}_i^{\vartheta }= \left\{ x\in {\mathbb {R}}: {\mathcal {N}}_{i0}^{\vartheta }(x)= \Vert {\mathcal {N}}_{i0}^{\vartheta }\Vert _\infty \right\} . \end{aligned}$$

The set ${\mathcal {S}}_i^{\vartheta }$ is referred to as the set of Evolutionary Attractors (or dominant strains) of the within-host model in the classical adaptive dynamics theory ( e.g., Geritz et al. 1997; Metz et al. 1996). Furthermore, when the function ${\mathcal {N}}_{i0}^{\vartheta }$ is at least of class ${\mathcal {C}}^1$, with a finite number of maximum, it is shown in Djidjou-Demasse et al. (2017) that these dominant strains coincide with the set ${\mathcal {S}}_i^{\vartheta }$. Denoting by $H^{\vartheta }_{i,\varepsilon }$, the operator $H^{\vartheta }_i$—by replacing the kernel *J* by $J_\varepsilon$—by results in Djidjou-Demasse et al. (2017) (Theorem 2.2), the spectral radius $r(H^{\vartheta }_{i,\varepsilon })$ of $H^{\vartheta }_{i,\varepsilon }$ satisfied, for $\varepsilon$ sufficiently small

$$\begin{aligned} r(H^{\vartheta }_{i,\varepsilon })= \left( {\mathcal {N}}_{i0}^{\vartheta }(x^*)\right) ^2 + {\mathcal {O}}(\varepsilon ), \quad \text {for all } x^* \in {\mathcal {S}}_i^{\vartheta }. \end{aligned}$$

By the above estimate, $\textrm{sign}\left[ r\left( H^{\vartheta }_{i,\varepsilon }\right) -1 \right] = \textrm{sign}\left[ {\mathcal {N}}_{i0}^{\vartheta }(x^*)-1 \right]$, for all $x^*\in$ and $\varepsilon$ sufficiently small. Furthermore, if $\varepsilon \ll 1$, ${\mathcal {S}}_i^{\vartheta }=\{x_i^*\}$ and $N_{i0}^{\vartheta }(x_i^*)>1$, then the unique positive stationary state ${\overline{F}}_{i}^{\vartheta }\equiv {\overline{F}}_{i,\varepsilon }^{\vartheta }$, given by (2.3), of the within-host model (1.1) is concentrated around the evolutionary attractor $x_i^*$ in the space of resistance level ${\mathbb {R}}$. In other words, $x_i^*$ is the average bacterial resistance level at the within-host scale equilibrium and we have $\lim _{\varepsilon \rightarrow 0}\int _{{\mathbb {R}}}u(x) {\overline{F}}_{i,\varepsilon }^{\vartheta }(x)\textrm{x}=u\left( x_i^*\right)$ for any continuous function $u \in {\mathcal {C}}\left( {\mathbb {R}}\right)$. We refer to Theorem 2.3 in Djidjou-Demasse et al. (2017) for such a concentration phenomenon.

### Key findings of the nested within- and between-host dynamics

At the between-host scale, by setting ${{\textbf {S}}}(t)=(S_i(t))_{i\in {\mathcal {I}}}$, ${{\textbf {I}}}_i(t,\tau )=(I_i^T(t,\tau ),I_i^U(t,\tau ))$, ${{\textbf {I}}}(t,\tau )=({{\textbf {I}}}_i(t,\tau ))_{i\in {\mathcal {I}}}$, $\varvec{\alpha }_i(\tau )= \text {diag}(\alpha _i^T(\tau ),\alpha _i^U(\tau ))$, $\varvec{\beta }_i(\tau )=(\beta _i^T(\tau ), \beta _i^U(\tau ))$, ${{\textbf {q}}}_i=(q_i^T,q_i^U)$, $$\varvec{\omega }(\tau )=\left( \begin{array}{cc} 0 &{} \omega _T^U(\tau ) \\ \omega _U^T(\tau ) &{} 0 \\ \end{array}\right) $$, and $${\varvec{e}}= \begin{pmatrix} 0 &{} 1\\ 1 &{} 0 \end{pmatrix} $$, System (1.4)–(1.5) rewrites into the following compact form,

2.5 $$\begin{aligned} {\left\{ \begin{array}{ll} \displaystyle \dot{{{\textbf {S}}}}(t)=\varvec{\Lambda }-{{\textbf {S}}}(t)\lambda (t) -\mu _h{{\textbf {S}}}(t),\\ \displaystyle {{\textbf {I}}}(t,\tau =0)=\lambda (t)\, \text {diag}({{\textbf {S}}}(t))\,{{\textbf {q}}},\\ \displaystyle \left( \partial _t+\partial _{\tau }\right) {{\textbf {I}}}(t,\tau )=\left( - \text {diag} (\varvec{\Phi }(\tau )) + \text {diag}(\varvec{\omega }(\tau )) \right) {{\textbf {I}}}(t,\tau ), \\ \end{array}\right. } \end{aligned}$$

where $$\lambda (t)=\sum _i\int _0^{\infty }\left\langle \varvec{\beta }_i(\tau ),{{\textbf {I}}}_i(t,\tau )\right\rangle \textrm{d}\tau $$, $$\varvec{\Lambda }=(\Lambda _i)_{i\in {\mathcal {I}}}$$, $${{\textbf {q}}}=({{\textbf {q}}}_i)_{i\in {\mathcal {I}}}$$, $$\varvec{\Phi }(\tau )=(\varvec{\Phi }_i(\tau ))_{i\in {\mathcal {I}}}$$, with $$\varvec{\Phi }_i(\tau )= {\varvec{e}}\varvec{\omega }(\tau ) +\varvec{\alpha }_i(\tau )+\mu _h$$.

Using the next-generation operator approach (e.g., Diekmann et al. 1990; Inaba 2012), the basic reproduction number ${\mathcal {R}}_0^i$ of the whole infected individuals of group *i*, is given by

$$\begin{aligned} {\mathcal {R}}_0^i= \frac{\Lambda _i}{\mu _h} \sum _{k\in {\mathcal {I}}} \int _0^\infty \left\langle \varvec{\beta }_k(\tau ), \varvec{\Pi }_k(\tau ,0) {{\textbf {q}}}_k \right\rangle \textrm{d}\tau =\frac{\Lambda _i}{\mu _h} \sum _{k\in {\mathcal {I}}} \chi _k, \end{aligned}$$

where

$$\begin{aligned} \chi _k = \int _0^\infty \left\langle \varvec{\beta }_k(\tau ), \varvec{\Pi }_k(\tau ,0) {{\textbf {q}}}_k \right\rangle \textrm{d}\tau , \end{aligned}$$

and where $\varvec{\Pi }_i(\tau _2,\tau _1)$, $0\le \tau _1\le \tau _2<\infty$, is the evolutionary system generated by the linear operator $\left[ -\varvec{\Phi }_i(\tau )+ \varvec{\omega }(\tau ) \right]$; see Remark 2.5 for some details on $\varvec{\Pi }_i$. Moreover, the basic reproduction number ${\mathcal {R}}_0$ at the whole between-host scale is such that

2.6 $$\begin{aligned} \displaystyle {\mathcal {R}}_0=&\sum _{i\in {\mathcal {I}}} \frac{\Lambda _i}{\mu _h} \chi _i = \sum _{i\in {\mathcal {I}}} \underbrace{\dfrac{\chi _i }{\sum _{k\in {\mathcal {I}}} \chi _k}}_{\begin{array}{c} \text {Overall relative infectiousness}\\ \text {of individuals of group }i. \end{array}} \times {\mathcal {R}}_0^i. \end{aligned}$$

We refer to Sect. 5.2 for details of the computation of ${\mathcal {R}}_0^i$,s and ${\mathcal {R}}_0$.

Note that the parameter $\chi _k$ quantifies the overall infectiousness of the whole infected individuals of group $k\in {\mathcal {I}}$. A more explicit expression of the infectiousness $\chi _k$ is difficult to obtain in general. However, one can go further steps in some particular configurations of the treatment status transition rates $\varvec{\omega }(\tau ).$ Indeed, assume that we can find $\tau _0>0$ and $\tau _1>0$ such that

2.7 $$\begin{aligned} \omega _T^U(\tau )= & {} {\left\{ \begin{array}{ll} 0, \quad \text {for} \quad \tau<\tau _0,\\ {\bar{\omega }}_1, \quad \text {for} \quad \tau _0<\tau<\tau _0+\tau _1, \end{array}\right. } \quad \text {and} \quad \nonumber \\ \omega ^T_U(\tau )= & {} 0, \quad \text {for}, \quad \tau <\tau _0+\tau _1. \end{aligned}$$

In the above scenario, the regimen $(0,\tau _0)$-post infection may corresponds to the initial phase where each infections, either treated or untreated, remain to their initial treatment status. The second regimen $(\tau _0,\tau _0+\tau _1)$-post infection may corresponds to the phase during which previously untreated infections becomes treated while treated infections remain to their initial status. In such a configuration, we have (see Sect. 5.2 for details)

$$\begin{aligned} \chi _k= \displaystyle q_k^T \chi _k^T + q_k^U \chi _k^U + {\mathcal {O}}\left( e^{-c_k (\tau _0+ \tau _1)} \right) , \end{aligned}$$

with $$c_k=\mu _h + \inf _\tau \alpha _k^U(\tau ) + \inf _\tau \alpha _k^T(\tau )$$,

$$\begin{aligned} \displaystyle \chi _k^T= & {} \int _0^{\tau _0+\tau _1} \beta _k^T(\tau ) \Gamma _{0}^{k,T} (\tau ) \textrm{d}\tau + \Gamma _{0}^{k,T} (\tau _0) {\bar{\omega }}_1 \int _{\tau _0}^{\tau _0+\tau _1} \beta _k^T(\tau ) \int _{\tau _0}^\tau \frac{\Gamma _{1}^{k,T} (\tau )}{\Gamma _{1}^{k,T} (\eta )} \Gamma _{1}^{k,U} (\eta ) \textrm{d} \eta \textrm{d}\tau ,\\ \displaystyle \chi _k^U= & {} \int _0^{\tau _0} \beta _k^U(\tau ) \Gamma _{0}^{k,U} (\tau ) \textrm{d}\tau + \Gamma _{0}^{k,U} (\tau _0) \int _{\tau _0}^{\tau _0+\tau _1} \beta _k^U(\tau ) \frac{\Gamma _{1}^{k,U} (\tau )}{\Gamma _{1}^{k,U} (\tau _0)} \textrm{d}\tau , \end{aligned}$$

and

$$\begin{aligned} \Gamma _{0}^{k,\vartheta } (\tau )= & {} e^{-\mu _h\tau } e^{-\int _0^\tau \alpha _k^\vartheta (\sigma ) \textrm{d}\sigma }, \quad \vartheta \in \{T,U\},\\ \Gamma _{1}^{k,U} (\tau )= & {} e^{-({\bar{\omega }}_1+\mu _h)\tau } e^{-\int _0^\tau \alpha _k^U(\sigma ) \textrm{d}\sigma },\quad \Gamma _{1}^{k,T} (\tau )= e^{-\mu _h\tau } e^{-\int _0^\tau \alpha _k^T (\sigma ) \textrm{d}\sigma }. \end{aligned}$$

Note that parameters $\Gamma _{0}^{k,\vartheta }$,s and $\Gamma _{1}^{k,\vartheta }$,s are survival probabilities during phases $(0,\tau _0)$ and $(\tau _0,\tau _0+\tau _1)$-post infection of infected individuals of group $k\in {\mathcal {I}}$, treated ($\vartheta =T$) or untreated ($\vartheta =U$).

#### Remark 2.5

Let $\varvec{\Pi }_i(\tau _2,\tau _1)$, $0\le \tau _1\le \tau _2<\infty$, the evolutionary system generated by the linear operator ${\varvec{A}}_i(\tau ):= -\varvec{\Phi }_i(\tau )+ \varvec{\omega }(\tau )$. It means that $\varvec{\Pi }_i$ is generated from the following evolutionary system

2.8 $$\begin{aligned} \left( \partial _t+\partial _{\tau }\right) I_i(t,\tau )= {\varvec{A}}_i(\tau ) I_i(t,\tau ). \end{aligned}$$

If, for example, the linear operator ${\varvec{A}}_i$ is diagonal, we have

$$\begin{aligned} I_i(t,\tau )= {\left\{ \begin{array}{ll} e^{\int _0^\tau {\varvec{A}}_i(\eta ) \textrm{d}\eta } I_i(t-\tau ,0)= \varvec{\Pi }_i(\tau ,0) I_i(t-\tau ,0); \quad t>\tau \\ e^{\int _{\tau -t}^\tau {\varvec{A}}_i(\eta ) \textrm{d}\eta } I_i(0,\tau -t)=\varvec{\Pi }_i(\tau ,\tau -t)I_i(0,\tau -t); \quad t<\tau . \end{array}\right. } \end{aligned}$$

In such a configuration we explicitly have $\varvec{\Pi }_i(\tau _2,\tau _1)= e^{\int _{\tau _1}^{\tau _2} {\varvec{A}}_i(\eta ) \textrm{d}\eta }.$ However, obtaining an explicit expression for $\varvec{\Pi }_i$ may not always be straightforward or possible in general. A naive approach would be to solve problem (2.8) as above, but it is well known that such an exponent formula does not give a solution to the problem at hand.

In addition to the disease-free equilibrium—the DFE—${{\textbf {E}}}^0=({{\textbf {S}}}^0,{{\textbf {0}}}_{L^1((0,\infty ),{\mathbb {R}}^{2n})})$, with ${{\textbf {S}}}^0=\left( \Lambda _i/\mu _h \right) _{i\in {\mathcal {I}}}$, which is always an equilibrium of Model (1.4)–(1.5), this model also exhibits an endemic equilibrium given by the following result

#### Theorem 2.6

Let Assumptions 2.1 and 2.2 hold. If ${\mathcal {R}}_0>1$, then system (1.4)–(1.5) has a unique endemic equilibrium ${{\textbf {E}}}^*=({{\textbf {S}}}^*,{{\textbf {I}}}^*(\tau ))$, such that $\forall i\in {\mathcal {I}}$,

$$\begin{aligned} S_i^*=\frac{S_i^0}{{\mathcal {R}}_0}\quad \text{ and }\quad {{\textbf {I}}}_i^*(\tau )=\lambda ^*S_i^* \; \varvec{\Pi }_i(\tau ,0)\; {{\textbf {q}}}_i, \end{aligned}$$

where $$\lambda ^*=\mu _h({\mathcal {R}}_0-1)$$.

Therefore, the threshold dynamics of Model (1.4)–(1.5) is summarized as follows

#### Theorem 2.7

Let Assumptions 2.1 and 2.2 hold. Then,

(i) If ${\mathcal {R}}_0\le 1$ or $\sum _{i\in {\mathcal {I}}}\int _0^{\infty } \left\langle \varvec{\beta }_i(\tau ),{{\textbf {I}}}_{i0}(\tau ) \right\rangle \textrm{d}\tau =0$, then the disease-free equilibrium ${{\textbf {E}}}^0=({{\textbf {S}}}^0,\,{{\textbf {0}}}_{L^1((0,\infty ),{\mathbb {R}}^{2n})})^t$ of system (1.4)-(1.5) is globally asymptotically stable in the sens that
  $$\begin{aligned} \lim _{t\rightarrow \infty } \left( S_i(t), I_i^T(t,\cdot ), I_i^U(t,\cdot ) \right) _{i \in {\mathcal {I}}} = {{\textbf {E}}}^0, \end{aligned}$$
  where the above convergence holds for the topology of ${\mathbb {R}}^n\times L^1((0,\infty ), {\mathbb {R}}^{2n})$.
(ii) If ${\mathcal {R}}_0>1$ and $\sum _{i\in {\mathcal {I}}}\int _0^{\infty } \left\langle \varvec{\beta }_i(\tau ),{{\textbf {I}}}_{i0}(\tau ) \right\rangle \textrm{d}\tau >0$, then the endemic equilibrium ${{\textbf {E}}}^*$ of system (1.4)-(1.5) is globally asymptotically stable, that is,
  $$\begin{aligned} \lim _{t\rightarrow \infty } \left( S_i(t), I_i^T(t,\cdot ), I_i^U(t,\cdot ) \right) _{i \in {\mathcal {I}}} = {{\textbf {E}}}^*, \end{aligned}$$
  for the topology of ${\mathbb {R}}^n\times L^1((0,\infty ), {\mathbb {R}}^{2n})$.

## Numerical illustrations

Here, we present a series of numerical simulations employing semi-explicit finite difference numerical schemes. We refer to Djidjou-Demasse (2021) for an example of a code repository within the context of the model proposed here. We illustrate an example of typical dynamics that can be simulated by the nested model (1.1)–(1.5). The model simultaneously captures the outbreak dynamics as well as the evolutionary dynamics of the average resistance level within the host population. The within-host model parameters are basically the same as in Djidjou-Demasse et al. (2023). Intuitively there exist two threshold levels, assumed here 0 and 1 (called reference “sensitive” and “resistant” strains) such that, a strain with resistance level *x* can be classically referred to as “sensitive”, “intermediate”, or “resistant” depending on whether $x < 0$, $0< x < 1$, or $x > 1$. For sake of simplicity, we assume that the host population is homogeneous in terms of immune system level, i.e., $\text {card}({\mathcal {I}})=1$. For all illustrative scenarios, we will have ${\mathcal {R}}_0>1$ such that the disease is persistent at the between-host scale (Theorem 2.7). The probability density function at the within-host scale ($J\equiv J_\varepsilon$) is assumed of type (2.4). Specifically, we define $J_\varepsilon$ as a Gaussian distribution $J_\varepsilon (x)=\frac{1}{\varepsilon \sqrt{2\pi }}e^{-\frac{1}{2}\left( \frac{x}{\varepsilon }\right) ^2}$, where $\varepsilon >0$ represents a small parameter that signifies the mutation variance within the phenotypic space.

*Within-host parameterization* The antimicrobial killing rate function $k(\cdot )$ is a decreasing function with respect to the resistance level *x* such that, $k(x)=k_0\left( \frac{k_1}{k_0}\right) ^x,$ where $k_0$ and $k_1$ are the antimicrobial activity undergone by the reference sensitive and resistant strains. Moreover, knowing $p_0$ and $p_1$, respectively the intrinsic growth rate of reference strains 0 and 1, a suitable expression for function of *p* is $p(x)= p_m \left[ 1+\left( \frac{p_m-p_0}{p_0}\right) \left( \frac{p_0}{p_1}\cdot \frac{p_m-p_1}{p_m-p_0} \right) ^x \right] ^{-1},$ where $p_m$ is the upper bound of the intrinsic growth rate *p* and $0<p_1<p_0<p_m$. The qualitative behaviour of functions *k* and *p* can be found in (Djidjou-Demasse et al. 2023, Fig. 2). We assume that the clearance rate of the bacteria cell due to the immune response, $\mu (\cdot )$, is a constant function given by $\mu (x)=\mu$. Furthermore, the average fitness cost-benefit ratio of resistance within a bacterial population can be expressed as $c_b=\frac{\log (\Delta )}{\log (1+\delta )}$. Here, $\Delta =\frac{(p_m-p_1)/p_1}{(p_m-p_0)/p_0}>1$ quantifies the relative cost of resistance, while $\delta =\frac{k_0-k_1}{k_1}>0$ measures the fitness advantage of the reference resistant strain (see Djidjou-Demasse et al. 2023 for details).

**Fig. 2:**
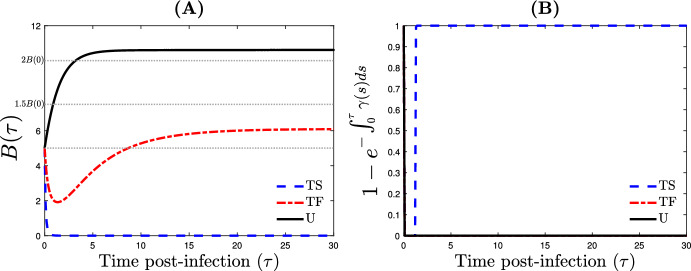
Illustration of the within- and between-host parameterization of infected individuals, either untreated—U, under treatment failure—TF, or under treatment with success—TS. **A** The total bacteria load $B^{\vartheta }(\tau )$. **B** The recovery probability $1-\exp (-\int _0^\tau \gamma ^\vartheta (s) \textrm{d}s)$

*Between-host parameterization* Parameters $\beta _i^{\vartheta }$ and $\alpha _i^{\vartheta }$ are defined using Holling type functional responses introduce by (1.2). For all simulations, the threshold $B_{min}$, introduced by (1.3), below which the infection becomes undetectable such that the infected individuals is considered as recovered is fixed as $B_{min}=10^{-3}B_{0}$, with $B_{0}=B(0)$ the initial total bacteria load. The total bacteria load ($B_i^{\vartheta }(\tau )$) and the recovery probability ($1-\exp (-\int _0^\tau \gamma _{i}^\vartheta (s) \textrm{d}s$), $\tau$-time post infection, are illustrated in Fig. 2.

Furthermore, an untreated infected individual joins the treated compartment when her total bacteria load is above a threshold $(1+\theta )\, B_{0}$, with $\theta \ge 0$. Therefore, the influx rate from untreated to treated is assumed to be a function with respect to time $\tau$ and is defined as follows

3.1 $$\begin{aligned} \omega _{T}^U(\tau )= \left\{ \begin{array}{ll} 0, &{} \hbox {if} \quad B^U(\tau )\le (1+\theta )\,B_{0}, \\ 1, &{} \hbox {if}\quad B^U(\tau )>(1+\theta )\,B_{0}. \end{array} \right. \end{aligned}$$

Similarly, we assume that an infected individual under treatment can drop down such a treatment when the bacteria load reach the same range as before the treatment. Therefore, the influx rate from treated to untreated is given by

3.2 $$\begin{aligned} \omega _{U}^T(\tau )= \left\{ \begin{array}{ll} 0, &{} \hbox {if} \quad B^T(\tau )\le B_{0}, \\ 1, &{} \hbox {if}\quad B^T(\tau )>B_{0}. \end{array} \right. \end{aligned}$$

*Initial conditions and model outputs* The initial bacterial population $b_0^{\vartheta }(x)$ is assumed to be composed by a sensitive bacterial population with average resistance level $x=0$. Hence, we set $b_0^{\vartheta }(x)=m_0\times {\mathcal {N}}(0,\sigma _0,x),$ where ${\mathcal {N}}(0,\sigma _0,x)$ stands for the normalized density function of the Gaussian distribution at *x* with mean 0 and variance $\sigma _0^2$. This means that the initial bacterial population is mostly composed of the reference “sensitive” strain. At the between-host scale, the initial condition of the epidemiological model is taken such that the susceptible population starts close to its disease-free equilibrium. More precisely, assuming an initial infection prevalence denoted as $\mathrm{P_{rev}}=10\%$, we derive the initial susceptible population as $S_{0}=(1-\mathrm{P_{rev}}) \frac{\Lambda }{\mu _h}$, along with the initial distribution of infectives which consists of $I_{0}^T(\tau )=0$ and $I_{0}^U(\tau )=\mathrm{P_{rev}} \frac{\Lambda }{\mu _h} \times L(\tau )$ for all $\tau \ge 0$. Here, $L(\tau )=10\ln (10)\times 10^{-10\tau }$, and it is important to note that *L* symbolizes the arbitrary initial distribution of individuals who have been infected since time $\tau$. This distribution is scaled so that $\int _{\mathbb {R}}L(\tau ) \textrm{d}\tau =1$.

The average level of resistance at within-host scale ($\eta (t)$) of the host population at time *t* is such that

$$\begin{aligned} \displaystyle \eta (t)= \int _0^{\infty } \left( \bar{x}^{T}(\tau )\; \frac{I^T(t,\tau )}{I(t)} + \bar{x}^{U}(\tau )\; \frac{I^U(t,\tau )}{I(t)} \right) \textrm{d}\tau , \end{aligned}$$

where $\bar{x}^{\vartheta }$,s are the individual average level of resistance and $I(t)=\int _0^{\infty } \big (I^T(t,\tau ) +I^U(t,\tau ) \big ) \textrm{d}\tau .$

*Simulated scenarios* Two simulated scenarios are considered, the first when the treatment is successful at the within-host level, and the second when the treatment failed at the within-host level. For all our simulated scenarios, the infection is assumed here to be always successful for untreated individuals, i.e., the immune system alone is no more enough to control the infection such that $\max _{x\in {\mathbb {R}}} {\mathcal {N}}_{0}^{U}(x) >1$, leading to the bacterial persistence for untreated infections.

**Fig. 3:**
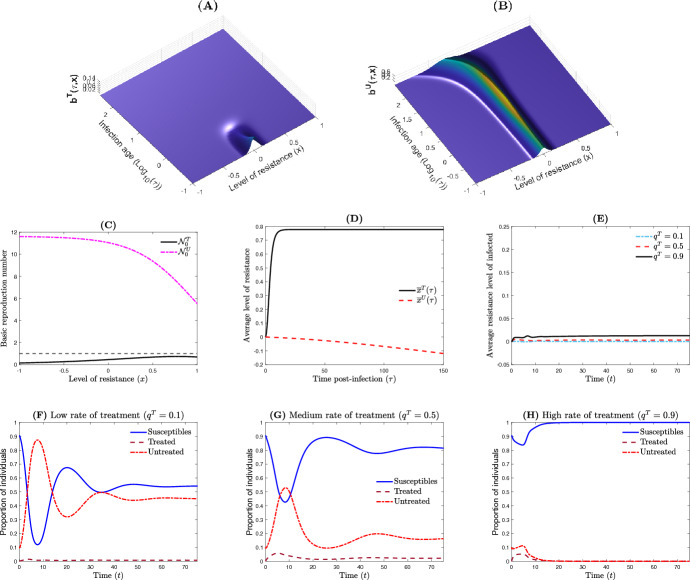
Dynamics of the nested model when the therapeutic treatment is successful. **A**, **B** The bacterial population (treated and untreated) with respect to time $\tau$ and resistance level *x*. **C** The basic reproduction numbers at the within-host level ${\mathcal {N}}_0^T(x)$ and ${\mathcal {N}}_0^U(x)$, with and without drug respectively. **D** The average resistance level $\bar{x}^{\vartheta }(\tau )$, $\vartheta \in \{T,U\}$ of the infected host. **E** The average resistance level $\eta (t)$ of the host population. **F**–**H** The between-host dynamics with respect to the treatment rate where ${\mathcal {R}}_0=4.7815$, 2.6750 and 0.5685, respectively. Parameter values are $(\sigma _0,m_0,k_0,p_1/p_0,k_1/k_0,\mu )= (0.05,0.05,20,0.5,0.3,0.8598)$ and $\Lambda =5\textrm{e}4$, $\mu _h=5.2675\hbox {e}{-}2$, $\beta _0=1.2\hbox {e}{-}3$, $\alpha _0=7.5\hbox {e}{-}2$, $\theta =1$, $r_0=9\textrm{e}3$

Our first scenario is for the case where the treatment is successful at the within-host level, i.e., the basic reproduction number of treated individuals ${\mathcal {N}}_{0}^{T}$ is such that $\max _{x\in {\mathbb {R}}} {\mathcal {N}}_{0}^{T}(x) <1$ (Fig. 3C). In such a situation, the bacterial load is under control in the relatively short term for treated individuals (Fig. 3A), while it remains persistent for untreated individuals (Fig. 3B). At the between-host scale, the treatment rate have a strong effect on the epidemic outbreak (Fig. 3F–H). More precisely, increasing the treatment rate $q^T$ in the host population strongly reduce the overall epidemic size (Fig. 3F–H), with ${\mathcal {R}}_0=4.7815$, 2.6750 and 0.5685, respectively. Furthermore, the average resistance level in the host population rapidly reach an equilibrium for which the level of resistance is moderately high compared to the initial resistance level of the host population (Fig. 3E).

**Fig. 4:**
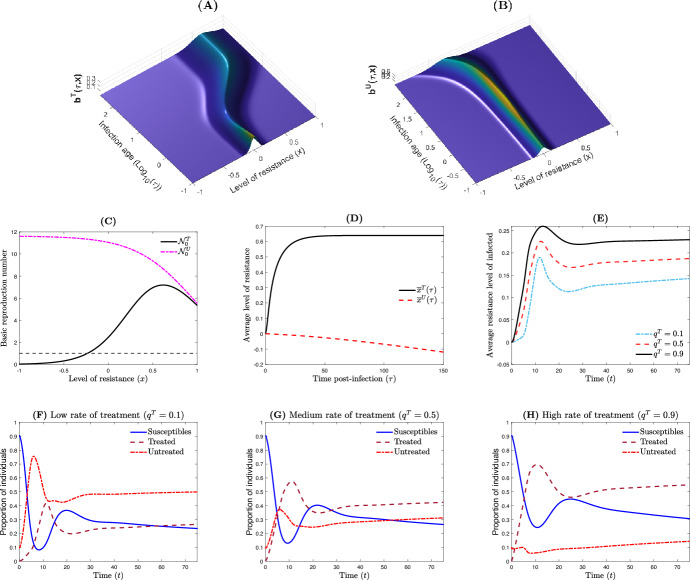
Dynamics of the nested model when the therapeutic treatment failed. **A**, **B** The bacterial population (treated and untreated) with respect to time $\tau$ and resistance level *x*. **C** The basic reproduction numbers at the within-host level ${\mathcal {N}}_0^T(x)$ and ${\mathcal {N}}_0^U(x)$, with and without drug respectively. **D** The average resistance level $\bar{x}^{\vartheta }(\tau )$, $\vartheta \in \{T,U\}$ of the infected host. **E** The average resistance level $\eta (t)$ of the host population. **F**–**H** The between-host dynamics with respect to the treatment rate where ${\mathcal {R}}_0=10.9451$, 9.5855 and 8.2259, respectively. Parameter values are $(\sigma _0,m_0,k_0,p_1/p_0,k_1/k_0,\mu )= (0.05,0.05,3,0.5,0.01,0.8598)$ and $\Lambda =5\hbox {e}4$, $\mu _h=5.2675\hbox {e}{-}2$, $\beta _0=1.2\hbox {e}{-}3$, $\alpha _0=7.5\hbox {e}{-}2$, $\theta =1$, $r_0=9\hbox {e}3$

**Fig. 5:**
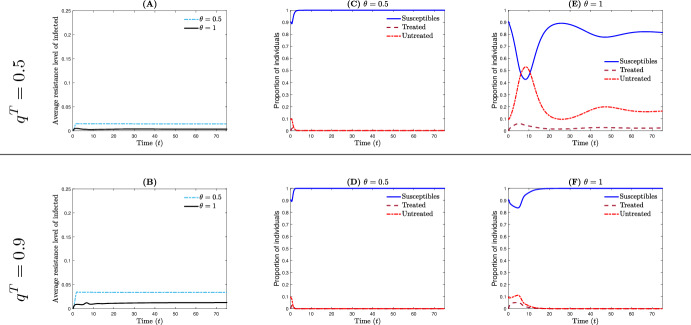
The effect of delay for treating infections on the epidemic outbreak and the average level of resistance when the therapeutic treatment is successful. *Line 1* The treatment rate $q^T=0.5$. **A** The average resistance level $\eta$ of the host population for $\theta \in \{0.5,1\}$. **C**, **E** The between-host dynamics for $\theta =0.5$ and 1, where ${\mathcal {R}}_0=0.3922$ and 2.6750 respectively. *Line 2* As in line 1 for $q^T=0.9$ where ${\mathcal {R}}_0=0.1120$ and 0.5685 respectively. Other parameter values are $(\sigma _0,m_0,k_0,p_1/p_0,k_1/k_0,\mu )= (0.05,0.05,20,0.5,0.3,0.8598)$ and $\Lambda =5\hbox {e}4$, $\mu _h=5.2675\hbox {e}{-}2$, $\beta _0=1.2\hbox {e}{-}3$, $\alpha _0=7.5\hbox {e}{-}2$, $r_0=9\hbox {e}3$

In the second scenario, the treatment is assumed unsuccessful at the within-host level, i.e., the basic reproduction number of treated individuals ${\mathcal {N}}_{0}^{T}$ is such that $\max _{x\in {\mathbb {R}}} {\mathcal {N}}_{0}^{T}(x) >1$ (Fig. 4C). In such a situation, the bacterial load remains persistent for both treated and untreated infections (Fig. 4A,B). Indeed, while we can observe an apparent decreasing of the bacteria load for some period of time for treated infections (Fig. 4A), at the end, we have the re-emergence of the bacteria population at within-host scale. Such a transient dynamics is explain by the fact that the initiation of treatment modifies the fitness landscape by shifting the maximum point of the within-host basic reproduction number ${\mathcal {N}}_{0}^{T}$ to the point $x = x^* > 0$ (Fig. 4C). In contrast to the treatment success scenario (Fig. 3), increasing the treatment rate $q^T$ in the host population have marginal effect in controlling the epidemic outbreak (Fig. 4F–H). In fact, with $q^T=0.1$, 0.5 and 0.9, the outbreak remains persistent with ${\mathcal {R}}_0=10.9451$, 9.5855 and 8.2259, respectively. Significantly, it is worth noting that although the average resistance level in the host population continues to rise with the treatment rate $q^T$ at equilibrium (Fig. 4E), there is a substantial increase in the range of resistance levels compared to the initial resistance level. This stands in contrast to the treatment success scenario (Fig. 3E), where the range of resistance levels remained relatively low. Additionally, in the treatment success scenario (Fig. 3E), the average resistance level in the host population quickly reaches equilibrium. However, in the treatment failure scenario (Fig. 4E), there is a comparatively longer transient period before the average resistance level in the host population reaches equilibrium. Overall, during the transient regimen, there is an initial subsequent increase in the average level of resistance to significantly higher levels, followed by a small decrease (Fig. 4E). This behavior is mostly attributed to the alteration of the fitness landscape caused by the treatment (Fig. 4C).

## Discussion

*Optimizing the treatment rate in the host population is key to controlling both the epidemic outbreak and the average level of resistance* Increasing the treatment rate in the host population contributes to reducing the epidemic size at the between-host scale, although the effect is quite marginal in the treatment failure scenario at the within-host scale (Figs. 3F–H, 4F–H). This can be primarily attributed to the fact that, even in cases of treatment failure, the within-host infection remains controlled for a certain period (Fig. 4A). However, the subsequent phase is characterized by an increase in bacterial population density (Fig. 4A). Conversely, raising the treatment rate within the host population leads to an elevation in the average resistance level of that population, regardless of the treatment scenario at the within-host scale (Figs. 3E, 4E). However, this increase in the average resistance level is particularly pronounced in the case of treatment failure (Fig. 4E), in comparison to the case of treatment success (Fig. 3E). In the case of treatment success, the resistance level remains highly similar to that of the initial bacterial population. Consequently, depending on the treatment regimen implemented within the host population, it becomes essential and intriguing to determine an optimal treatment rate to effectively manage both the outbreak and the average level of antimicrobial resistance.

*The delay for treating infections can impact the epidemic outbreak as well as average level of resistance* Assume the scenario where the treatment is successful at the within-host scale (Fig. 3A) and at least 50% of infected individuals are under treatment ($q^T\ge 0.5$). Two configurations are introduced. In the first configuration, untreated individuals begin treatment at a rate $\omega ^U_T$ [defined by (3.1)] when their total bacterial load reaches a threshold value of $1.5\times B_0$, i.e., $\theta =0.5$. In the second configuration, untreated individuals start treatment at a rate $\omega ^U_T$ when their total bacterial load reaches a threshold value of $2\times B_0$, i.e., $\theta =1$. The case of $\theta =0.5$ indicates a situation where the delay before initiating the treatment is very short. On the other hand, in the case of $\theta =1$, the delay before starting the treatment is relatively more significant (Fig. 2A). In general, the average resistance level in the host population decreases as the delay before initiating treatment increases (Fig. 5A, B). One possible explanation is that untreated infected individuals do not significantly contribute to the increase in the average resistance level within the host population. Nevertheless, although early treatment effectively controls the epidemic outbreak for both moderate and high treatment rates (Fig. 5C, D)—with ${\mathcal {R}}_0=0.3922$ and 0.1120, respectively—delaying the treatment of infected individuals leads to an epidemic that is out of control, except in cases where the treatment rate is exceptionally high (Fig. 5E, F)—with ${\mathcal {R}}_0=2.6750$ and 0.5685, respectively.

*Nested models parameterization issue* Explicitly connecting the within- to and between-host scales are crucial to gain a more realistic picture aiming to integrate into the same modelling framework the epidemic dynamics and the evolutionary dynamics of antimicrobial resistance. However, such an approach requires making assumptions about the parameters at the within-host scale that are equally unknown as the parameters at the between-host scale and hence leading to uncertainty about the appropriate parametrization (Uecker and Bonhoeffer 2021). Such uncertainty is amplified by our approach where the level of resistance is considered a continuous quantitative trait, compared to the classical qualitative (or “binary”) approaches (Djidjou-Demasse et al. 2023).

*Time-scale separation hypothesis* Within the context of nesting within- and between-host scales, for the modelling of the epidemiology and evolution of pathogens, some studies assumed that the epidemiological and evolutionary time scales are distinct, i.e., the within-host dynamic is fast relative to the between-host dynamic such that the within-host model remains at equilibrium, e.g. (Gilchrist and Coombs 2006; Xue and Bloom 2020; Almocera et al. 2018; Boldin and Diekmann 2008; Coombs et al. 2007; André and Gandon 2006). Although such an assumption on the within-host equilibrium dynamic’ might be appropriate for chronic infections, it leads to a population-scale model that does not explicitly account for the individual time-dependent infectiousness dynamics (Hart et al. 2020). Furthermore, our illustrative examples (Figs. 3, 4) strongly highlight the infectiousness’ time-dependency of infected individuals and show that the between-host dynamic is not necessarily faster compared to the within-host dynamic.

## Preliminaries and technical materials

We will go through details on the proof of our main results, namely Theorem 2.7. We will first discuss the existence of a positive global solution of the nested model. Next, we will give details on the derivation of the basic reproduction number of System (1.4)–(1.5) for individuals with an immune system of level *i*. We will also derive the existence of a unique endemic equilibrium of System (1.4)–(1.5) when ${\mathcal {R}}_0>1$, as well as the long-term persistence of the epidemic in such a case.

### Existence of the semiflow

We establish the existence of a positive global solution of the system (2.5). We first formulate system (2.5) in an abstract Cauchy problem. For that, we introduce the Banach space ${\mathcal {X}}={\mathbb {R}}^n\times {\mathbb {R}}^{2n} \times L^1((0,\infty ), {\mathbb {R}}^{2n})$, endowed with the usual product norm $\Vert \cdot \Vert _{{\mathcal {X}}}$ as well as its positive cone ${\mathcal {X}}_+$. Let $A\,:\, D(A)\subset {\mathcal {X}}\rightarrow {\mathcal {X}}$ be the linear operator defined by $D(A)={\mathbb {R}}^n\times \{{{\textbf {0}}}_{{\mathbb {R}}^{2n}}\}\times W^{1,1}((0,\infty ),{\mathbb {R}}^{2n})$ and

5.1 $$\begin{aligned} A({{\textbf {S}}},{{\textbf {0}}}_{{\mathbb {R}}^{2n}},{{\textbf {I}}})=\Big (-\mu _h{{\textbf {S}}},\!-{{\textbf {I}}}(0),\!-\partial _{\tau }{{\textbf {I}}}+ \left( - \text {diag} (\varvec{\Phi }(\tau )) + \text {diag}(\varvec{\omega }(\tau )) \right) {{\textbf {I}}} \Big ).\qquad \end{aligned}$$

Let us introduce the non-linear map $F\;:\; \overline{D(A)}\rightarrow {\mathcal {X}}$ defined by

$$\begin{aligned} F({{\textbf {S}}},{{\textbf {0}}}_{{\mathbb {R}}^{2n}},{{\textbf {I}}})= \left( \varvec{\Lambda }-{{\textbf {S}}}(t)\lambda (t),\, \lambda (t)\, \text {diag}({{\textbf {S}}}(t))\,{{\textbf {q}}}, \text {diag}(\varvec{\omega }(\cdot )){{\textbf {I}}} \right) . \end{aligned}$$

By identifying $\varvec{\varphi }(t)$ together with $({{\textbf {S}}}(t),{{\textbf {0}}}_{L^1},{{\textbf {I}}}(t,\cdot ))^t$ and by setting $\varvec{\varphi }_{0}=({{\textbf {S}}}_0,{{\textbf {0}}}_{L^1},{{\textbf {I}}}_0(\cdot ))^t$ the associated initial condition, system (2.5) becomes

5.2 $$\begin{aligned} \left\{ \begin{array}{lll} \displaystyle \frac{\textrm{d}\varvec{\varphi }(t)}{\textrm{d}t}=A\varvec{\varphi }(t)+F(\varvec{\varphi }(t)),\\ \varvec{\varphi }(0)=\varvec{\varphi }_{0}. \end{array} \right. \end{aligned}$$

By setting ${\mathcal {X}}_0 = D(A)$ and ${\mathcal {X}}_{0+} = {\mathcal {X}}_0 \cap {\mathcal {X}}_+$, the positivity and boundedness of the solutions of system (2.5) are provided by the following result.

#### Theorem 5.1

There exists a unique strongly continuous semiflow $\big \{ \varvec{\Psi }(t,\cdot ): {\mathcal {X}}_0 \rightarrow {\mathcal {X}}_0\big \}_{t\ge 0}$ such that, for each $\varvec{\varphi }_{0} \in {\mathcal {X}}_{0+}$, the map $\varvec{\varphi } \in {\mathcal {C}} \left( [0,\infty ), {\mathcal {X}}_{0+} \right)$ defined by $\varvec{\varphi }=\varvec{\Psi }(\cdot ,\varvec{\varphi }_{0})$ is a mild solution of (5.2). That is, $\int _0^t \varvec{\varphi }(s) \textrm{d} s \in D(A)$ and $\varvec{\varphi }(t)= \varvec{\varphi }_{0} + A \int _0^t \varvec{\varphi }(s)d s + \int _0^t F\left( \varvec{\varphi }(s)\right) \textrm{d} s$ for all $t\ge 0$. Moreover, $\left\{ \varvec{\Psi }(t,\cdot ) \right\} _t$ satisfies the following properties:

1. Let $\varvec{\Psi }(t,\varvec{\varphi }_{0})=\left( {{\textbf {S}}}(t),{{\textbf {0}}}_{{\mathbb {R}}^{2n}},{{\textbf {I}}}(t,\cdot ) \right) ^t$, then the following Volterra formulation holds true for all $i\in {\mathcal {I}}$
  5.3 $$\begin{aligned} {{\textbf {I}}}_i(t,\tau )=\left\{ \begin{array}{ll} \displaystyle \varvec{\Pi }_i(\tau ,\tau -t)\, {{\textbf {I}}}_{i0}(\tau -t), &{} \hbox {if} \quad t\le \tau , \\ \displaystyle \lambda (t-\tau )\;S_i(t-\tau )\;\varvec{\Pi }_i(\tau ,0)\, {{\textbf {q}}}_i, &{} \hbox {if}\quad t>\tau , \end{array} \right. \end{aligned}$$
  coupled with the $S_i(t)$ equation of (2.5), and where $\varvec{\Pi }_i(\tau _2,\tau _1)$, $0\le \tau _1\le \tau _2<\infty$, is the evolutionary system generated by the linear operator $\left[ -\varvec{\Phi }_i(\tau )+ \varvec{\omega }(\tau ) \right]$.
2. For all $\varvec{\varphi }_{0} \in {\mathcal {X}}_{0+}$, and for all $t\ge 0$, one has
  5.4 $$\begin{aligned} \sum _{i\in {\mathcal {I}}}\left( S_i(t) + \int _0^\infty \Big (I_i^T(t,\tau )+ I_i^U(t,\tau ) \Big )\textrm{d}\tau \right) \le \max \left\{ \frac{{\overline{\Lambda }}}{\mu _h},N_0 \right\} , \end{aligned}$$
  where ${\overline{\Lambda }}=\sum _{i\in {\mathcal {I}}}\Lambda _i$ and $N_0= \sum _{i\in {\mathcal {I}}}\left( S_{i0} + \int _0^\infty \Big (I_{i0}^T(\tau )+ I_{i0}^U(\tau ) \Big )\textrm{d}\tau \right)$. Furthermore, the subset of the phase space
  $$\begin{aligned} \left\{ ({{\textbf {S}}},{{\textbf {I}}})\in {\mathbb {R}}^n\times L^1{((0,\infty ),{\mathbb {R}}^{2n})}\, \Big |\, \displaystyle \sum _{i\in {\mathcal {I}}}\left( S_i(t) + \int _0^\infty \Big (I_i^T(t,\tau )+ I_i^U(t,\tau ) \Big )\textrm{d}\tau \right) \le \frac{{\overline{\Lambda }}}{\mu _h} \right\} , \end{aligned}$$
  is positively invariant and attracts all nonnegative solutions.
3. The semiflow $\left\{ \varvec{\Psi }(t,\cdot ) \right\} _t$ generated by (2.5) is bounded dissipative, that is, there exists a bounded set ${\mathcal {B}} \subset {\mathcal {X}}_{0}$ such that for any bounded set $U \subset {\mathcal {X}}_{0}$, we can find $\sigma = \sigma (U,{\mathcal {B}}) \ge 0$ such that $\varvec{\Psi }(t,U) \subset {\mathcal {B}}$ for $t\ge \sigma$.

#### Proof

It is easy to check that the operator *A* is a Hille-Yosida operator. Then standard results apply to provide the existence and uniqueness of a mild solution to (2.5) [we refer to Magal and Ruan (2009) and Thieme (2011) for more details]. The Volterra formulation is also standard and we refer to Iannelli (1995), Webb (1985) for more details.

The $S_i$ equation of (2.5) gives $\dot{S}_i(t) \le \Lambda _i-\mu _{h} S_i(t)$, that is

$$\begin{aligned} S_i(t) \le \max \left\{ \frac{\Lambda _i}{\mu _h}, S_{i0} \right\} . \end{aligned}$$

Next, for estimate (5.4), let $\varvec{\varphi }_{0} \in {\mathcal {X}}_{0+}$, then adding up the $S_i$,s equation together with the $I_i^T$,s and $I_i^U$,s equations of (1.5) yields for all $i\in {\mathcal {I}}$

$$\begin{aligned}{} & {} \frac{d}{dt} \left( S_i(t) + \int _{{\mathbb {R}}}\int _0^\infty \Big (I_i^T(t,\tau )+ I_i^U(t,\tau ) \Big )\textrm{d}\tau \right) \\{} & {} \quad \le \Lambda _i- \mu _hS_i(t)- \mu _h\int _{{\mathbb {R}}}\int _0^\infty \Big (I_i^T(t,\tau )+ I_i^U(t,\tau ) \Big )\textrm{d}\tau . \end{aligned}$$

It comes

$$\begin{aligned} \displaystyle \limsup \limits _{t\rightarrow \infty }\sum _{i\in {\mathcal {I}}} \left( S_i(t) + \int _{{\mathbb {R}}}\int _0^\infty (I_i^T(t,\tau )+ I_i^U(t,\tau ))\textrm{d}\tau \right) \le \frac{{\overline{\Lambda }}}{\mu _h}, \end{aligned}$$

with ${\overline{\Lambda }}= \sum _{i\in {\mathcal {I}}} \Lambda _i$. From where one deduces estimate (5.4) and which ends item 2. of the theorem.

The bounded dissipativity of the semiflow $\left\{ \varvec{\Psi }(t,\cdot ) \right\} _t$ is a direct consequence of estimate 2. $\square$

The following result is straightforward.

#### Lemma 5.2

Let $0\le \tau _1\le \tau _2<\infty$. By setting $\displaystyle \varvec{\Pi }_i(\tau _2,\tau _1)= \Big (\Pi _{i,j}^k(\tau _2,\tau _1)\Big )_{k,j}$, with $k,j\in \{T,U\}$, we have

5.5 $$\begin{aligned} \gamma _0 e^{-(\mu _h+\alpha _0)(\tau _2-\tau _1)} \le \Pi _{i,j}^k(\tau _2,\tau _1)\le \gamma _1 e^{-\mu _h(\tau _2-\tau _1)}, \end{aligned}$$

where $$\gamma _0$$, $$\gamma _1>0$$ and $$\alpha _0= \max _i(\sup \alpha _i^T,\sup \alpha _i^U)$$.

#### Proof

Let $0\le \tau _1\le \tau _2<\infty$. Note that, for all $\tau \ge 0$, we have $-\omega _1\le \omega _U^T(\tau )\le \omega _1$ and $-\omega _2\le \omega ^U_T(\tau )\le \omega _2$, with $\omega _i>0$. Then, for all $\tau \ge 0$,

$$\begin{aligned} \begin{pmatrix} -\omega _1-\alpha _0-\mu _h &{} \omega _2 \\ \omega _1 &{} -\omega _2-\alpha _0-\mu _h \end{pmatrix} \le -\varvec{\Phi }_i(\tau )+ \varvec{\omega }(\tau )\le \begin{pmatrix} -\omega _1 -\mu _h &{} \omega _2 \\ \omega _1 &{} -\omega _2-\mu _h \end{pmatrix}. \end{aligned}$$

Therefore,

$$\begin{aligned} e^{-(\mu _h+\alpha _0)(\tau _2-\tau _1)} \varvec{\Gamma }(\tau _2,\tau _1) \le \varvec{\Pi }_i(\tau _2,\tau _1)\le e^{-\mu _h(\tau _2-\tau _1)} \varvec{\Gamma }(\tau _2,\tau _1), \end{aligned}$$

where

$$\begin{aligned}\displaystyle \varvec{\Gamma }(\tau _2,\tau _1)=\begin{pmatrix} \displaystyle \frac{\omega _1e^{-(\tau _2-\tau _1)(\omega _1+\omega _2)}+\omega _2}{\omega _1 +\omega _2} &{} \displaystyle \frac{\omega _2}{\omega _1+\omega _2} \left( 1-e^{-(\tau _2-\tau _1)(\omega _1+\omega _2)} \right) \\ \displaystyle \frac{\omega _1}{\omega _1+\omega _2} \left( 1-e^{-(\tau _2-\tau _1)(\omega _1+\omega _2)} \right) &{} \displaystyle \frac{\omega _2e^{-(\tau _2-\tau _1)(\omega _1+\omega _1)}+\omega _2}{\omega _1 +\omega _2} \end{pmatrix}. \end{aligned}$$

Note that

$$\begin{aligned} \underline{\varvec{\Gamma }} \le \varvec{\Gamma }(\tau _2,\tau _1)\le \overline{\varvec{\Gamma }},\quad \text{ where }\quad \overline{\varvec{\Gamma }}= \begin{pmatrix} 1 &{} \frac{\omega _2}{\omega _1+\omega _2} \\ \frac{\omega _1}{\omega _1+\omega _2} &{} 1 \end{pmatrix}\quad \text{ and }\quad \underline{\varvec{\Gamma }}= \begin{pmatrix} \frac{\omega _2}{\omega _1+\omega _2} &{} 0 \\ 0 &{} \frac{\omega _1}{\omega _1+\omega _2} \end{pmatrix}.\end{aligned}$$

Thus,

$$\begin{aligned} e^{-(\mu _h+\alpha _0)(\tau _2-\tau _1)} \underline{\varvec{\Gamma }} \le \varvec{\Pi }_i(\tau _2,\tau _1)\le e^{-\mu _h(\tau _2-\tau _1)} \overline{\varvec{\Gamma }}, \end{aligned}$$

from where inequality (5.5) follows, and this ends the proof of the lemma. $\square$

### The basic reproduction number

In the absence of infection, that is ${{\textbf {I}}}(t,\tau )={{\textbf {0}}}_{L^1((0,\infty ),{\mathbb {R}}^{2n})}$, the system (2.5) has a disease-free equilibrium (DFE) given by ${{\textbf {E}}}^0=({{\textbf {S}}}^0,{{\textbf {0}}}_{L^1((0,\infty ),{\mathbb {R}}^{2n})})$, with ${{\textbf {S}}}^0=\left( \Lambda _i/\mu _h \right) _{i\in {\mathcal {I}}}$. Let $\Theta _i(t)$ be the number of new infections in the host population of group *i* at time *t*. Then in an initially infection-free population, by (2.5), we have

$$\begin{aligned} \Theta _i(t) = \lambda (t)\;S_i^0 = \left( \sum _{k\in {\mathcal {I}}}\int _0^{\infty } \left\langle \varvec{\beta }_k(\tau ), {{\textbf {I}}}_k(t,\tau ) \right\rangle \textrm{d}\tau \right) \; S_i^0, \end{aligned}$$

where $\left\langle \cdot ; \cdot \right\rangle$ is the usual scalar product.

Linearizing the Volterra formulation (5.3) at the DFE, it comes

$$\begin{aligned} {{\textbf {I}}}_k(t,\tau )=\left\{ \begin{array}{ll} \varvec{\Pi }_k(\tau ,\tau -t)\,{{\textbf {I}}}_{k0}(\tau -t), &{} \hbox {if}\quad t\le \tau , \\ \\ \lambda (t-\tau )\, S_k^0\, \varvec{\Pi }_k(\tau ,0) {{\textbf {q}}}_k, &{} \hbox {if}\quad t>\tau . \end{array} \right. \end{aligned}$$

From where,

$$\begin{aligned} \Theta _i(t)&= S_i^0 \sum _{k\in {\mathcal {I}}}\int _0^t \lambda (t-\tau ) S_k^0 \left\langle \varvec{\beta }_k(\tau ), \varvec{\Pi }_k(\tau ,0)\, {{\textbf {q}}}_k \right\rangle \textrm{d}\tau +f_i(t),\quad \forall t\ge 0 ,\\&= S_i^0 \sum _{k\in {\mathcal {I}}}\int _0^t \left\langle \varvec{\beta }_k(\tau ), \varvec{\Pi }_k(\tau ,0)\, {{\textbf {q}}}_k \right\rangle \Theta _k(t-\tau ) \textrm{d}\tau +f_i(t),\quad \forall t\ge 0 , \end{aligned}$$

where $f_i(t)$ is the number of new infections produced by the initial population. Therefore, the basic reproduction number ${\mathcal {R}}_0^i$ of individuals of group *i* is calculated as

$$\begin{aligned} {\mathcal {R}}_0^i= S_i^0 \sum _{k\in {\mathcal {I}}}\int _0^\infty \left\langle \varvec{\beta }_k(\tau ), \varvec{\Pi }_k(\tau ,0)\, {{\textbf {q}}}_k \right\rangle \textrm{d}\tau =S_i^0\sum _{k\in {\mathcal {I}}}\chi _k, \end{aligned}$$

with

$$\begin{aligned} \chi _k = \int _0^\infty \left\langle \varvec{\beta }_k(\tau ), \varvec{\Pi }_k(\tau ,0) {{\textbf {q}}}_k \right\rangle \textrm{d}\tau . \end{aligned}$$

The term $\chi _k(\tau )= \left\langle \varvec{\beta }_k(\tau ), \varvec{\Pi }_k(\tau ,0) {{\textbf {q}}}_k \right\rangle$ quantifies the infectiousness at $\tau$-time post infection of the whole infected individuals of group $k \in {\mathcal {I}}$.

Next, let $\Theta (t)=(\Theta _i(t))_{i\in {\mathcal {I}}}$, the number of new infections in all groups at time *t* and, $f(t)=(f_i(t))_{i\in {\mathcal {I}}}$ the number of new infections produced by the initial population. We have

$$\begin{aligned} \Theta (t)&=\left( S_i^0 \sum _{k\in {\mathcal {I}}}\int _0^t \chi _k(\tau ) \Theta _k(t-\tau ) \textrm{d}\tau \right) _{i\in {\mathcal {I}}} + f(t) \\&= \left( \int _{0}^{t} \left( S_i^0 \chi _k(\tau ) \right) _{i,k\in {\mathcal {I}}} \Theta (t-\tau )\textrm{d}\tau \right) + f(t),\quad \forall t>0, \end{aligned}$$

Due to the above formulation, the basic reproduction number ${\mathcal {R}}_0$ of all individuals is calculated as the spectral radius of the matrix $(a_{i,k})_{i,k\in {\mathcal {I}}}$, where

$$\begin{aligned} a_{i,k}=S_i^0\int _{0}^{\infty } \chi _k(\tau ) \textrm{d}\tau =S_i^0\;\chi _k. \end{aligned}$$

Some calculations give

$$\begin{aligned} {\mathcal {R}}_0=&\sum _{k\in {\mathcal {I}} }S_k^0\int _0^\infty \chi _k(\tau ) \textrm{d}\tau =\sum _{k\in {\mathcal {I}} } S_k^0\;\chi _k. \end{aligned}$$

A more explicit expression of the infectiousness $\chi _k$ is difficult to obtain in general. However, one can go further steps in some particular configurations of the treatment status transition rates $$\varvec{\omega }(\tau )=\left( \begin{array}{cc} 0 &{} \omega _T^U(\tau ) \\ \omega _U^T(\tau ) &{} 0 \\ \end{array}\right) .$$ Indeed, assume that (2.7) holds. In such a configuration, we have

$$\begin{aligned} \varvec{\Pi }_k(\tau ,0)= \left\{ \begin{array}{ll} \varvec{\Pi }_{k0}(\tau ,0), &{} \hbox {if} \quad 0<\tau<\tau _0, \\ \varvec{\Pi }_{k0}(\tau _0,0) \varvec{\Pi }_{k1}(\tau ,\tau _0), &{} \hbox {if}\quad \tau _0<\tau <\tau _0+\tau _1,\\ {\mathcal {O}}\left( e^{-c_k \tau } \right) , &{} \hbox {if} \quad \tau > \tau _0+\tau _1, \end{array} \right. \end{aligned}$$

with $$c_k=\mu _h + \inf _\tau \alpha _k^U(\tau ) + \inf _\tau \alpha _k^T(\tau )$$, and

$$\begin{aligned} \varvec{\Pi }_{k0}(\tau ,0)= & {} \displaystyle e^{-\mu _h\tau } \text {diag} \left( e^{-\int _0^\tau \alpha _k^T(\sigma ) \textrm{d}\sigma }, e^{-\int _0^\tau \alpha _k^U(\sigma ) \textrm{d}\sigma } \right) , \\ \varvec{\Pi }_{k1}(\tau ,0)= & {} \textrm{diag} \left( {\bar{\omega }}_1 \int _0^\tau e^{-\mu _h(\tau -\eta )} e^{-\int _\eta ^\tau \alpha _k^T(\sigma ) \textrm{d}\sigma } e^{-({\bar{\omega }}_1+\mu _h)\eta } e^{-\int _0^\eta \alpha _k^U(\sigma ) \textrm{d}\sigma } \right. \\{} & {} \left. \textrm{d} \eta , e^{-({\bar{\omega }}_1+\mu _h)\tau } e^{-\int _0^\tau \alpha _k^U(\sigma ) \textrm{d}\sigma } \right) . \end{aligned}$$

From where, by setting

$$\begin{aligned} \Gamma _{0}^{k,\vartheta } (\tau )= & {} e^{-\mu _h\tau } e^{-\int _0^\tau \alpha _k^\vartheta (\sigma ) \textrm{d}\sigma }, \quad \vartheta \in \{T,U\},\\ \Gamma _{1}^{k,U} (\tau )= & {} e^{-({\bar{\omega }}_1+\mu _h)\tau } e^{-\int _0^\tau \alpha _k^U(\sigma ) \textrm{d}\sigma },\\ \Gamma _{1}^{k,T} (\tau )= & {} e^{-\mu _h\tau } e^{-\int _0^\tau \alpha _k^T (\sigma ) \textrm{d}\sigma }, \end{aligned}$$

it comes

$$\begin{aligned} \chi _k= \displaystyle q_k^T \chi _k^T + q_k^U \chi _k^U + {\mathcal {O}}\left( e^{-c_k (\tau _0+ \tau _1)} \right) , \end{aligned}$$

where

$$\begin{aligned} \displaystyle \chi _k^T= & {} \int _0^{\tau _0+\tau _1} \beta _k^T(\tau ) \Gamma _{0}^{k,T} (\tau ) \textrm{d}\tau + \Gamma _{0}^{k,T} (\tau _0) {\bar{\omega }}_1 \int _{\tau _0}^{\tau _0+\tau _1} \beta _k^T(\tau ) \int _{\tau _0}^\tau \frac{\Gamma _{1}^{k,T} (\tau )}{\Gamma _{1}^{k,T} (\eta )} \Gamma _{1}^{k,U} (\eta ) \textrm{d} \eta \textrm{d}\tau ,\\ \displaystyle \chi _k^U= & {} \int _0^{\tau _0} \beta _k^U(\tau ) \Gamma _{0}^{k,U} (\tau ) \textrm{d}\tau + \Gamma _{0}^{k,U} (\tau _0) \int _{\tau _0}^{\tau _0+\tau _1} \beta _k^U(\tau ) \frac{\Gamma _{1}^{k,U} (\tau )}{\Gamma _{1}^{k,U} (\tau _0)} \textrm{d}\tau . \end{aligned}$$

### Proof of Theorem 2.6

The equilibrium of system (2.5) is obtained by solving the following system for all $i\in {\mathcal {I}}$

5.6 $$\begin{aligned} {\left\{ \begin{array}{ll} 0=\Lambda _i-S_i^*\lambda ^*-\mu _h S_i^*,\\ \displaystyle {{\textbf {I}}}_i^*(\tau =0)=\lambda ^*S_i^*\;{{\textbf {q}}}_i,\\ \displaystyle \partial _{\tau } {{\textbf {I}}}_i^*(\tau )= (-{\varvec{e}}\varvec{\omega }(\tau )- \varvec{\alpha }_i(\tau )-\mu _h+ \varvec{\omega }(\tau )){{\textbf {I}}}_i^*(\tau ), \\ \end{array}\right. } \end{aligned}$$

where

5.7 $$\begin{aligned} \displaystyle \lambda ^*= \sum _{i\in {\mathcal {I}}}\int _0^{\infty }\left\langle \varvec{\beta }_i(\tau ), {{\textbf {I}}}_i^*(\tau )\right\rangle \textrm{d}\tau . \end{aligned}$$

Solving (5.6) for $S_i^*$ and ${{\textbf {I}}}_i^*$ yields

5.8 $$\begin{aligned} S_i^*=\dfrac{\Lambda _i}{\mu _h+\lambda ^*}\quad \text{ and }\quad {{\textbf {I}}}_i^*(\tau )= \lambda ^*\;S_i^*\; \varvec{\Pi }_i(\tau ,0)\; {{\textbf {q}}}_i. \end{aligned}$$

Replacing (5.8) in (5.7) leads to $\left( 1+\mu _h^{-1}\lambda ^*\right) \lambda ^*= {\mathcal {R}}_0 \lambda ^*$ and since $\lambda ^*>0$, we have $\lambda ^*=\mu _h({\mathcal {R}}_0-1)$.

It follows that system (2.5) has a unique positive endemic equilibrium when ${\mathcal {R}}_0>1$, such that $\forall i\in {\mathcal {I}}$,

$$\begin{aligned} S_i^*=\frac{S_i^0}{{\mathcal {R}}_0}\quad \text{ and }\quad {{\textbf {I}}}_i^*(\tau )=\lambda ^*S_i^* \; \varvec{\Pi }_i(\tau ,0)\; {{\textbf {q}}}_i, \end{aligned}$$

where $$\lambda ^*=\mu _h({\mathcal {R}}_0-1)$$.

### Technical materials

Before proceed to the proof Theorem 2.7, we introduce some technical materials including the existence of a global compact attractor for the solution semiflow of Model (2.5), the spectral properties of the linearized semiflow of Model (2.5) at any given equilibrium, and the uniform persistence of Model (2.5) when ${\mathcal {R}}_0>1$.

#### Global compact attractor

To derive the global properties of the solution dynamics, it is necessary to show that the semiflow generated by system (2.5) has a global compact attractor. Denote by

$$\begin{aligned} {\mathcal {Y}}={\mathbb {R}}^n\times L^1((0,\infty ), {\mathbb {R}}^{2n})\quad \text{ and }\quad {\mathcal {Y}}_+={\mathbb {R}}^n_+\times L^1_+((0,\infty ), {\mathbb {R}}^{2n}), \end{aligned}$$

and endow the set ${\mathcal {Y}}$ with the norm

$$\begin{aligned} \Vert ({{\textbf {S}}},{{\textbf {I}}}(\cdot ))\Vert =\sum _{i\in {\mathcal {I}}}\left( |S_i|+\int _{0}^{\infty } \Big (|I_i^T(t,\tau )|+|I_i^U(t,\tau )|\Big )\textrm{d}\tau \right) . \end{aligned}$$

For any initial condition $\varvec{\varphi }_{0}\in {\mathcal {Y}}$, the solution semiflow of system (2.5) in ${\mathcal {Y}}_+$ is denoted by $\displaystyle \varvec{\Psi }^*(t,\varvec{\varphi }_{0})=({{\textbf {S}}}(t),{{\textbf {I}}}(t,\cdot ))^t$. From the Volterra formulation (5.3), we rewrite system (2.5) as follows for all $i\in {\mathcal {I}}$:

5.9 $$\begin{aligned} \displaystyle \dot{S}_i(t)= & {} \Lambda _i-S_i(t)\lambda (t) -\mu _h S_i(t), \nonumber \\ I^T_i(t,\tau )= & {} \left\{ \begin{array}{ll} \displaystyle \Pi ^T_{i,T}(\tau ,\tau -t)\, I^T_{i0}(\tau -t)+ \Pi ^T_{i,U}(\tau ,\tau -t)\, I^U_{i0}(\tau -t), &{} \hbox {if} \quad t\le \tau , \\ \displaystyle \Pi ^T_{i}(\tau ,0) \lambda (t-\tau )\;S_i(t-\tau ), &{} \hbox {if}\quad t>\tau , \end{array} \right. \qquad \end{aligned}$$

5.10 $$\begin{aligned} I^U_i(t,\tau )= & {} \left\{ \begin{array}{ll} \displaystyle \Pi ^U_{i,T}(\tau ,\tau -t)\, I^T_{i0}(\tau -t)+ \Pi ^U_{i,U}(\tau ,\tau -t)\, I^U_{i0}(\tau -t), &{} \hbox {if} \quad t\le \tau , \\ \displaystyle \Pi ^U_{i}(\tau ,0) \lambda (t-\tau )\;S_i(t-\tau ), &{} \hbox {if}\quad t>\tau , \end{array} \right. \qquad \end{aligned}$$

where

$$\begin{aligned} \Pi ^T_{i}(\tau ,0)=\Pi ^T_{i,T}(\tau ,0)q^T_i+ \Pi ^T_{i,U}(\tau ,0)q^U_i\quad \text{ and }\quad \Pi ^U_{i}(\tau ,0)=\Pi ^U_{i,T}(\tau ,0)q^T_i+ \Pi ^U_{i,U}(\tau ,0)q^U_i. \end{aligned}$$

We need to prove the following claim.

##### Claim 5.3

Let Assumption 2.2 be satisfied. Then, function $\lambda (\cdot )$ is Lipschitz continuous on ${\mathbb {R}}_+$.

##### Proof of Claim 5.3

Let $C_0\ge \max \left\{ \frac{{\overline{\Lambda }}}{\mu _h},\Vert \varvec{\varphi }_0\Vert \right\}$, $\Vert \beta _i\Vert _{\infty }=\max \Big \{\Vert \beta _i^T\Vert _{\infty }, \Vert \beta _i^U\Vert _{\infty }\Big \}$ and $\Vert \beta \Vert _{\infty }=\max _{i\in {\mathcal {I}}}\Vert \beta _i\Vert _{\infty }$. Then, $|\lambda (t)|\le C_0\Vert \beta \Vert _{\infty }$. Let $t>0$ and $h>0$. It comes that

$$\begin{aligned} \lambda (t+h)-\lambda (t)= & {} \sum _{i\in {\mathcal {I}}}\int _0^{h} \left\langle \varvec{\beta }_i(\tau ),{{\textbf {I}}}_{i}(t+h,\tau ) \right\rangle \textrm{d}\tau + \sum _{i\in {\mathcal {I}}}\int _h^{\infty } \left\langle \varvec{\beta }_i(\tau ),{{\textbf {I}}}_{i}(t+h,\tau ) \right\rangle \textrm{d}\tau \\{} & {} -\, \sum _{i\in {\mathcal {I}}}\int _0^{\infty } \left\langle \varvec{\beta }_i(\tau ),{{\textbf {I}}}_{i}(t,\tau ) \right\rangle \textrm{d}\tau \\= & {} \sum _{i\in {\mathcal {I}}}\int _0^{h} \left\langle \varvec{\beta }_i(\tau ),\varvec{\Pi }_i(\tau ,0) {{\textbf {I}}}_{i}(t+h-\tau ,0)\right\rangle \textrm{d}\tau \\{} & {} +\, \sum _{i\in {\mathcal {I}}}\int _h^{\infty } \left\langle \varvec{\beta }_i(\tau ),{{\textbf {I}}}_{i}(t+h,\tau ) \right\rangle \textrm{d}\tau - \,\sum _{i\in {\mathcal {I}}}\int _0^{\infty } \left\langle \varvec{\beta }_i(\tau ),{{\textbf {I}}}_{i}(t,\tau ) \right\rangle \textrm{d}\tau \\\le & {} C_0^2\Vert \beta \Vert ^2_{\infty }h+ \sum _{i\in {\mathcal {I}}}\int _0^{\infty } \left\langle \varvec{\beta }_i(\tau +h),{{\textbf {I}}}_{i}(t+h,\tau +h)\right\rangle \textrm{d}\tau \\{} & {} - \,\sum _{i\in {\mathcal {I}}}\int _0^{\infty } \left\langle \varvec{\beta }_i(\tau ),{{\textbf {I}}}_{i}(t,\tau ) \right\rangle \textrm{d}\tau . \end{aligned}$$

Recalling (5.3) and combining the integrals, we obtain

$$\begin{aligned} \lambda (t+h)-\lambda (t)\le & {} C_0^2\Vert \beta \Vert ^2_{\infty }h+ \sum _{i\in {\mathcal {I}}}\int _0^{\infty } \left\langle \varvec{\beta }_i(\tau +h), (\varvec{\Pi }_i(\tau +h,\tau )-{\mathbb {I}}){{\textbf {I}}}_{i}(t,\tau )\right\rangle \textrm{d}\tau \\{} & {} +\, \sum _{i\in {\mathcal {I}}}\int _0^{\infty } \left\langle \varvec{\beta }_i(\tau +h)- \varvec{\beta }_i(\tau ),{{\textbf {I}}}_{i}(t,\tau ) \right\rangle \textrm{d}\tau \\\le & {} C_0^2\Vert \beta \Vert ^2_{\infty }h+ \sum _{i\in {\mathcal {I}}}\int _0^{\infty } \left\langle \varvec{\beta }_i(\tau +h), (\gamma _1e^{-\mu _hh}-1){{\textbf {I}}}_{i}(t,\tau )\right\rangle \textrm{d}\tau \\{} & {} +\, \sum _{i\in {\mathcal {I}}}\int _0^{\infty } \left\langle \varvec{\beta }_i(\tau +h)- \varvec{\beta }_i(\tau ),{{\textbf {I}}}_{i}(t,\tau ) \right\rangle \textrm{d}\tau . \end{aligned}$$

We have $|\gamma _1e^{-\mu _hh}-1|\le |e^{-\mu _hh}-1|\le \mu _hh$. Using the Lipschitzianity of $\beta _i^{\vartheta }$, we find a positive constant $C_{\beta }$ such that

$$\begin{aligned} |\lambda (t+h)-\lambda (t)|\le & {} C_0^2\Vert \beta \Vert ^2_{\infty }h+ C_0\Vert \beta \Vert _{\infty }\mu _hh+C_{\beta }C_0h\le C_{\lambda }h, \end{aligned}$$

where $$C_{\lambda }=C_0^2\Vert \beta \Vert ^2_{\infty }+C_0 \Vert \beta \Vert _{\infty }\mu _h+C_{\beta }C_0$$. $$\square $$

Next, we will show that system (2.5) has a global attractor. By using the similar method as in Martcheva and Thieme (2003) and Cheng et al. (2018), we can state the following result.

##### Lemma 5.4

There exists ${\mathcal {A}}_{0}$, a compact subset of ${\mathcal {Y}}_+$, which is a global attractor for the solution semiflow of system (2.5). Moreover, ${\mathcal {A}}_{0}$ is invariant under the solution semiflow, that is

$$\begin{aligned} \varvec{\Psi }^*(t,\varvec{\varphi }_{0})\subseteq {\mathcal {A}}_{0},\quad \text{ for } \text{ every }\quad \varvec{\varphi }_{0}\in {\mathcal {A}}_{0},\quad \forall t\ge 0. \end{aligned}$$

##### Proof

We show that $\varvec{\Psi }^*$ satisfies the assumptions of Lemma 3.2.3 and Theorem 3.4.6 in Hale (2010). To this end, we split the solution semiflow into two parts. For any initial condition $\varvec{\varphi }_{0}\in {\mathcal {Y}}_+$, we let $\varvec{\Psi }^*(t,\varvec{\varphi }_{0})=\widehat{\varvec{\Psi }}^*(t,\varvec{\varphi }_{0})+ \widetilde{\varvec{\Psi }}^*(t,\varvec{\varphi }_{0})$, where

$$\begin{aligned} \widehat{\varvec{\Psi }}^*(t,\varvec{\varphi }_{0})=\left\{ \begin{array}{ll} \displaystyle ({{\textbf {0}}}_{{\mathbb {R}}^n},{{\textbf {0}}}_{L^1}), &{} t>\tau , \\ \displaystyle ({{\textbf {0}}}_{{\mathbb {R}}^n},{{\textbf {I}}}(t,\tau )), &{} t\le \tau , \end{array} \right. \quad \text{ and }\quad \widetilde{\varvec{\Psi }}^*(t,\varvec{\varphi }_{0})=\left\{ \begin{array}{ll} \displaystyle ({{\textbf {S}}}(t),{{\textbf {I}}}(t,\tau )), &{} t>\tau , \\ \displaystyle ({{\textbf {S}}}(t),{{\textbf {0}}}_{L^1}), &{} t\le \tau . \end{array} \right. \end{aligned}$$

In such a way, we need to prove the following claim:

##### Claim 5.5

1. $\widehat{\varvec{\Psi }}^*(t,\varvec{\varphi }_{0})\rightarrow 0$ as $t\rightarrow \infty$ for every $\varvec{\varphi }_{0}$ in ${\mathcal {Y}}$.
2. For a fixed *t* and any bounded set *B* in ${\mathcal {Y}}$, the set $\{\widetilde{\varvec{\Psi }}^*(t,\varvec{\varphi }_{0})\;:\; \varvec{\varphi }_{0}\in B\}$ is precompact.

##### Proof of Claim 5.5

Now, we show that the first claim holds.

From (5.9) and Lemma 5.2, we have

$$\begin{aligned} \Vert \widehat{\varvec{\Psi }}^*(t,\varvec{\varphi }_{0})\Vert= & {} \sum _{i\in {\mathcal {I}}} \int _{0}^{\infty }\Big (|I_i^T(t,\tau )| +|I_i^U(t,\tau )|\Big ) \textrm{d}\tau \\= & {} \sum _{i\in {\mathcal {I}}}\int _{0}^{\infty } [\Pi ^T_{i,T}(\tau ,\tau -t) +\Pi ^U_{i,T}(\tau ,\tau -t)]I^T_{i0}(\tau -t)\textrm{d}\tau \\{} & {} +\, \sum _{i\in {\mathcal {I}}}\int _{{\mathbb {R}}}\int _{0}^{\infty } [\Pi ^T_{i,U}(\tau ,\tau -t) +\Pi ^U_{i,U}(\tau ,\tau -t)]I^U_{i0}(\tau -t)\textrm{d}\tau \\\le & {} 2\gamma _1 e^{-\mu _ht}\Vert \varvec{\varphi }_{0}\Vert , \end{aligned}$$

Note that for any bounded $\varphi _{0}$, $2\gamma _1 e^{-\mu _ht} \Vert \varvec{\varphi }_{0}\Vert \rightarrow 0$ as $t\rightarrow \infty$. This completes the first claim.

To show that the second claim holds, let $B\subset {\mathcal {Y}}$ be a bounded subset such that $\varvec{\Psi }^*(t,\cdot )B\subset B$. Choose $C_0>0$ such that $\Vert \varvec{\varphi }_{0}\Vert \le C_0$ for all $\varvec{\varphi }_{0}\in B$. From Theorem 5.1 Item **2**, $\cup _{\varvec{\varphi }_{0}\in B}\{{{\textbf {S}}}(t)\}$ is bounded in ${\mathbb {R}}^n$ and then is precompact in ${\mathbb {R}}^n$. Hence, to show the compactness, it suffices to show that the set $\widetilde{\varvec{\Psi }}^*(t,\varvec{\varphi }_{0})B$ is precompact for

5.11 $$\begin{aligned} \widetilde{{{\textbf {I}}}}(t,\tau )=\left\{ \begin{array}{ll} \displaystyle {{\textbf {I}}}(t,\tau ), &{} t>\tau , \\ \displaystyle {{\textbf {0}}}_{L^1}, &{} t\le \tau . \end{array} \right. \end{aligned}$$

By Frechet–Kolmogorov theorem [see Theorem B.2 in Smith and Thieme (2011)], it is sufficient to verify the following conditions:

(i) $$\sup \limits _{\varvec{\varphi }\in B}\sum _{i\in {\mathcal {I}}}\int _{0}^{\infty }\Big ({\widetilde{I}}_i^T(t,\tau )+{\widetilde{I}}_i^U(t,\tau )\Big )\textrm{d}\tau <\infty $$,
(ii) $\lim \limits _{h\rightarrow \infty }\sum _{i\in {\mathcal {I}}}\int _{h}^{\infty } \Big (|{\widetilde{I}}_i^T(t,\tau )|+ |{\widetilde{I}}_i^U(t,\tau )|\Big )\textrm{d}\tau =0$ uniformly with respect to $\varvec{\varphi }_{0}\in B$.
(iii) $\lim \limits _{h\rightarrow 0}\sum _{i\in {\mathcal {I}}}\int _{0}^{\infty } \Big (|{\widetilde{I}}_i^T(t,\tau )-{\widetilde{I}}_i^T(t,\tau +h)|+ |{\widetilde{I}}_i^U(t,\tau )-{\widetilde{I}}_i^U(t,\tau +h)|\Big )\textrm{d}\tau =0$ uniformly with respect to $\varvec{\varphi }_{0}\in B$.
(iv) $\lim \limits _{h\rightarrow 0}\sum _{i\in {\mathcal {I}}}\int _{0}^{h} \Big (|{\widetilde{I}}_i^T(t,\tau )|+ |{\widetilde{I}}_i^U(t,\tau )|\Big )\textrm{d}\tau =0$ uniformly with respect to $\varvec{\varphi }_{0}\in B$.

By (5.11) we have for all $i\in {\mathcal {I}}$

$$\begin{aligned} \widetilde{{{\textbf {I}}}}_i(t,\tau )=\left\{ \begin{array}{ll} \lambda (t-\tau )\, S_i(t-\tau )\, \varvec{\Pi }_i(\tau ,0) {{\textbf {q}}}_i, &{} t>\tau , \\ {{\textbf {0}}}_{L^1}, &{} t\le \tau . \end{array} \right. \end{aligned}$$

It follows that above conditions (i), (ii) and (iv) are satisfied.

Now, we show that condition (*iii*) holds. We have for $i\in {\mathcal {I}}$ and $h\le t$,

$$\begin{aligned}{} & {} \int _{0}^{\infty } |{\widetilde{I}}_i^T(t,\tau )-{\widetilde{I}}_i^T(t,\tau +h)| \textrm{d}\tau \\{} & {} \quad =\int _{0}^{t-h}\Big |\Pi ^T_{i}(\tau ,0) \lambda (t-\tau )S_i(t-\tau )- \Pi ^T_{i}(\tau +h,0) \lambda (t-\tau -h)S_i(t-\tau -h)\Big |\textrm{d}\tau \\{} & {} \qquad +\,\int _{t-h}^{t}\Big |\Pi ^T_{i}(\tau ,0) \lambda (t-\tau )S_i(t-\tau )\Big |\textrm{d}\tau \\{} & {} \quad := C_1(t,h)+C_2(t,h). \end{aligned}$$

By Lemma 5.2, and the boundedness of the semiflow, we can find a positice constant $C_0$ such that

5.12 $$\begin{aligned} C_2(t,h)=\int _{t-h}^{t}\Big |\Pi ^T_{i}(\tau ,0) \lambda (t-\tau )S_i(t-\tau )\Big |\textrm{d}\tau \le C_0^2\Vert \beta \Vert _{\infty }\gamma _1 h \rightarrow 0\quad \text{ as }\quad h\rightarrow 0. \nonumber \\ \end{aligned}$$

Again by Lemma 5.2, we have

5.13 $$\begin{aligned} C_1(t,h)\le & {} \int _{0}^{t-h} \Pi ^T_{i}(\tau ,0) S_i(t-\tau ) \Big | \lambda (t-\tau )- \lambda (t-\tau -h)\Big |\textrm{d}\tau \nonumber \\{} & {} +\, \int _{0}^{t-h} \lambda (t-\tau -h) \Big |\Pi ^T_{i}(\tau ,0) S_i(t-\tau )- \Pi ^T_{i}(\tau +h,0)S_i(t-\tau -h)\Big |\textrm{d}\tau \nonumber \\\le & {} \int _{0}^{t-h} \Pi ^T_{i}(\tau ,0) S_i(t-\tau ) \Big | \lambda (t-\tau )- \lambda (t-\tau -h)\Big |\textrm{d}\tau \nonumber \\{} & {} + \,\int _{0}^{t-h}\lambda (t-\tau -h)S_i(t-\tau ) \Big |\Pi ^T_{i}(\tau ,0)-\Pi ^T_{i}(\tau +h,0)\Big |\textrm{d}\tau \nonumber \\{} & {} +\,\int _{0}^{t-h}\lambda (t-\tau -h)\Pi ^T_{i}(\tau +h,0)\Big |S_i(t-\tau )-S_i(t-\tau -h)\Big |\textrm{d}\tau \nonumber \\\le & {} C_0^2\Vert \beta \Vert _{\infty }\int _{0}^{\infty } \Big |\Pi ^T_{i}(\tau ,0)-\Pi ^T_{i}(\tau +h,0)\Big |\textrm{d}\tau \nonumber \\{} & {} +\,\gamma _1C_0 \int _{0}^{t-h}e^{-\mu _h\tau }\Big | \lambda (t-\tau )-\lambda (t-\tau -h)\Big | \textrm{d}\tau \nonumber \\{} & {} +\, \gamma _1C_0\Vert \beta \Vert _{\infty } \int _{0}^{t-h}e^{-\mu _h\tau } \Big |S_i(t-\tau )-S_i(t-\tau -h)\Big |\textrm{d}\tau \nonumber \\\le & {} C_0^2\Vert \beta \Vert _{\infty }\left| \partial _{\tau } \Pi ^T_{i}(\tau ,0) \right| |h|+\Big (\gamma _1C_0C_{\lambda } +\gamma _1C_0C_{S_i}\Vert \beta \Vert _{\infty }\Big )h\int _{0}^{t-h}e^{-\mu _h\tau } \textrm{d}\tau \nonumber \\\le & {} C_0^2\Vert \beta \Vert _{\infty }\left| \partial _{\tau } \Pi ^T_{i}(\tau ,0) \right| |h|+ \frac{\gamma _1C_0}{\mu _h}\Big (C_{\lambda } +C_{S_i}\Vert \beta \Vert _{\infty }\Big )h\rightarrow 0\quad \text{ as }\quad h\rightarrow 0, \nonumber \\ \end{aligned}$$

with $C_{S_i}=\Lambda _i+C_{i0}(C_0\Vert \beta \Vert _{\infty }+\mu _h)$. By (5.12) and (5.13) one concludes that the criterion (*iii*) holds, and then the second claim holds.

This completes the proof of the lemma. $\square$

#### Spectral properties of the linearized semiflow

The next result is concerned with spectral properties of the linearized semiflow $\varvec{\Psi }$ of Model (2.5) at a given equilibrium point $\widetilde{\varvec{\varphi }}\in {\mathcal {X}}_{0+}$. The associated linearized system (2.5) at the point $\widetilde{\varvec{\varphi }}$ reads as

$$\begin{aligned} \frac{d\varvec{\varphi }(t)}{dt}= (A+G[\widetilde{\varvec{\varphi }}])\varvec{\varphi }(t), \end{aligned}$$

where *A* is the linear operator defined in (5.1) while $G[\widetilde{\varvec{\varphi }}]\in {\mathcal {L}}({\mathcal {X}}_{0},{\mathcal {X}})$ is the bounded linear operator defined by:

$$\begin{aligned} G[\widetilde{\varvec{\varphi }}]\varvec{\varphi }=\left( \begin{array}{c} \displaystyle -{{\textbf {S}}}{\widetilde{\lambda }}-\widetilde{{{\textbf {S}}}} \lambda \\ \displaystyle {\widetilde{\lambda }}\, \text {diag}({{\textbf {S}}})\,{{\textbf {q}}}+ \lambda \,\text {diag}(\widetilde{{{\textbf {S}}}})\,{{\textbf {q}}}\\ \displaystyle {{\textbf {0}}}_{L^1((0,\infty ),{\mathbb {R}}^{2n})} \end{array} \right) , \end{aligned}$$

where ${\widetilde{\lambda }}=\sum _i\int _0^{\infty } \left\langle \varvec{\beta }_i(\tau ), \widetilde{{{\textbf {I}}}}_i(\tau )\right\rangle \textrm{d}\tau$ and $\lambda =\sum _i\int _0^{\infty } \left\langle \varvec{\beta }_i(\tau ), {{\textbf {I}}}_i(\tau )\right\rangle \textrm{d}\tau$. We then have the following lemma.

##### Lemma 5.6

Let us set $\Omega =\{\nu \in {\mathbb {C}}\,: \, Re(\nu )>-\mu _h\}$. Then, the spectrum $\sigma (A+G[\widetilde{\varvec{\varphi }}])\cap \Omega \ne \emptyset$ only consists of the point spectrum and one has

$$\begin{aligned} \sigma (A+G[\widetilde{\varvec{\varphi }}])\cap \Omega =\{\nu \in \Omega \,: \, \Delta (\nu ,\varvec{{\widetilde{\varphi }}})=0\}, \end{aligned}$$

where function $\Delta (\cdot ,\varvec{{\widetilde{\varphi }}}):\Omega \longrightarrow {\mathbb {C}}$ is defined by

5.14 $$\begin{aligned} \displaystyle \Delta (\nu ,\varvec{{\widetilde{\varphi }}})=1- {\mathcal {R}}_{\nu } [\varvec{{\widetilde{\varphi }}}] + \frac{{\mathcal {R}}_\nu [\widetilde{\varvec{\varphi }}]\,{\widetilde{\lambda }}}{\nu +\mu _h+{\widetilde{\lambda }}}, \end{aligned}$$

with $${\mathcal {R}}_\nu [\widetilde{\varvec{\varphi }}]=\sum _{i\in {\mathcal {I}}} {\widetilde{S}}_i\; \int _0^{\infty } \left\langle \varvec{\beta }_i(\tau ), \varvec{\Pi }_i(\tau ,0)\;{{\textbf {q}}}_i \right\rangle e^{-\nu \tau }\textrm{d}\tau . $$

##### Proof

Let us denote by $A_{0}\,:\, D(A_{0})\subset {\mathcal {X}}_0\rightarrow {\mathcal {X}}_0$ the part of *A* in ${\mathcal {X}}_0=D(A)$, which is defined by

$$\begin{aligned} A_{0}\varvec{\varphi }=A\varvec{\varphi },\quad \forall \varvec{\varphi }\in D(A_{0})=\{\varvec{\varphi }\in D(A)\,:\, A\varvec{\varphi }\in D(A)\}. \end{aligned}$$

Then, it is the infinitesimal generator of a $C_0$-semigroup on ${\mathcal {X}}_0$ denoted by $\{T_{A_{0}}(t)\}_{t\ge 0}$. Let $\varvec{\varphi }=({{\textbf {S}}},{{\textbf {0}}}_{{\mathbb {R}}^{2n}},{{\textbf {I}}}(\cdot ))^t$. We find that

$$\begin{aligned} T_{A_{0}}(t)\varvec{\varphi }(\tau )=\left\{ \begin{array}{ll} \displaystyle \Big (e^{-\mu _ht}{{\textbf {S}}},{{\textbf {0}}}_{{\mathbb {R}}^{2n}}, \varvec{\Pi }(\tau ,\tau -t)\, {{\textbf {I}}}(\tau -t)\Big )^t, &{} \forall t\le \tau , \\ \displaystyle \Big (e^{-\mu _ht}{{\textbf {S}}},{{\textbf {0}}}_{{\mathbb {R}}^{2n}}, {{\textbf {0}}}_{L^1((0,\infty ),{\mathbb {R}}^{2n})}\Big )^t, &{} \forall t>\tau . \end{array} \right. \end{aligned}$$

Then, for $t\ge \tau _0$, we have $\Vert T_{A_{0}}(t-\tau _0)\varvec{\varphi }\Vert _{{\mathcal {X}}}\le e^{-\mu _h(t-\tau _0)}\Vert \varvec{\varphi }\Vert _{{\mathcal {X}}}, \forall t\ge \tau _0.$ We deduce that the growth rate $\omega _0(A_{0})=\lim \limits _{t\rightarrow +\infty } \frac{\ln \Big (\Vert T_{A_{0}}(t)\Vert _{{\mathcal {L}}({\mathcal {X}})}\Big )}{t}$ of this semigroup satisfies $\omega _0(A_{0})\le -\mu _h.$ Since operator $G_i[\widetilde{\varvec{\varphi }}]$ is compact, the results in Arino et al. (1998) or Ducrot et al. (2008) apply and provided that the essential growth rate of $\Big \{ T_{(A+G[\widetilde{\varvec{\varphi }}])_0}(t)\Big \}_{t\ge 0}$-the $C_0$-semigroup generated by the part of $(A+G[\widetilde{\varvec{\varphi }}])$ in ${\mathcal {X}}_0$ satisfies

$$\begin{aligned} \omega _{0,ess}((A+G[\widetilde{\varvec{\varphi }}])_0)\le \omega _{0,ess}(A_{0})<\omega _{0}(A_{0})\le -\mu _h. \end{aligned}$$

By results in Engel and Nagel (2001) and Webb (1987), the latter inequality ensures that $\Omega \cap \sigma (A+G[\widetilde{\varvec{\varphi }}])\ne \emptyset$, and it is only composed of point spectrum of $(A+G[\widetilde{\varvec{\varphi }}])$.

It remains to derive the characteristic equation. Let $\nu \in \rho (A+G[\widetilde{\varvec{\varphi }}])$, where $\rho (\cdot )$ stands for the resolvent. For $\widehat{\varvec{\varphi }}= (\widehat{{{\textbf {S}}}},\widehat{{{\textbf {u}}}}, \widehat{{{\textbf {I}}}}(\cdot ))^t\in {\mathcal {X}}$ and $\varvec{\varphi }= ({{\textbf {S}}},0_{L^1}, {{\textbf {I}}}(\cdot ))^t\in D(A)$, we have $(\nu I-A-G[\widetilde{\varvec{\varphi }}])\varvec{\varphi }=\widehat{\varvec{\varphi }}$, that is $(\nu I- A)\varvec{\varphi }-G[\widetilde{\varvec{\varphi }}]\varvec{\varphi }=\widehat{\varvec{\varphi }}$, and from where

5.15 $$\begin{aligned} \varvec{\varphi }=(\nu I- A)^{-1}\widehat{\varvec{\varphi }}+(\nu I- A)^{-1} G[\widetilde{\varvec{\varphi }}]\varvec{\varphi }. \end{aligned}$$

Since

$$\begin{aligned} (\nu I- A)^{-1}\widehat{\varvec{\varphi }}= \left( \left( \frac{{\widehat{S}}_i}{\nu +\mu _h}\right) _{i\in {\mathcal {I}}},\,{{\textbf {0}}}_{{\mathbb {R}}^{2n}},\, \left( e^{-\nu \cdot }\varvec{\Pi }_i(\cdot ,0)\widehat{{{\textbf {u}}}}_i+ \int _{0}^{\cdot } \varvec{\Pi }_i(\cdot ,s)\widehat{{{\textbf {I}}}}_i(s) e^{-\nu (\cdot -s)}\textrm{d}s\right) _{i\in {\mathcal {I}}}\right) ^t, \end{aligned}$$

we find that

$$\begin{aligned} (\nu I- A)^{-1} G[\widetilde{\varvec{\varphi }}]\varvec{\varphi }=\left( \begin{array}{c} \displaystyle \left( \frac{-S_i{\widetilde{\lambda }}-{\widetilde{S}}_i \lambda }{\nu +\mu _h}\right) _{i\in {\mathcal {I}}} \\ {{\textbf {0}}}_{{\mathbb {R}}^{2n}}\\ \displaystyle \left( e^{-\nu \cdot }\varvec{\Pi }_i(\cdot ,0) \left( S_i{\widetilde{\lambda }}\;{{\textbf {q}}}_i+ {\widetilde{S}}_i\lambda \;{{\textbf {q}}}_i\right) \right) _{i\in {\mathcal {I}}}\\ \end{array} \right) . \end{aligned}$$

Thus, for all $i\in {\mathcal {I}}$, equality (5.15) rewrites as

5.16 $$\begin{aligned}{} & {} \displaystyle \left( 1+\frac{{\widetilde{\lambda }}}{\nu +\mu _h}\right) S_i +\frac{{\widetilde{S}}_i \lambda }{\nu +\mu _h} =\frac{{\widehat{S}}_i}{\nu +\mu _h},\nonumber \\{} & {} \quad {\left\{ \begin{array}{ll} \displaystyle S_i=\frac{{\widehat{S}}_i}{\nu +\mu _h+{\widetilde{\lambda }}}- \frac{{\widetilde{S}}_i \lambda }{\nu +\mu _h+{\widetilde{\lambda }}}\\ {{\textbf {I}}}_i(\tau )=e^{-\nu \tau }\varvec{\Pi }_i(\tau ,0) \left( S_i{\widetilde{\lambda }}{{\textbf {q}}}_i+ {\widetilde{S}}_i\lambda {{\textbf {q}}}_i\right) + e^{-\nu \tau }\varvec{\Pi }_i(\tau ,0)\widehat{{{\textbf {u}}}}_i\\ \qquad \qquad + \int _{0}^{\tau } \varvec{\Pi }_i(\tau ,s)\widehat{{{\textbf {I}}}}_i(s) e^{-\nu (\tau -s)}\textrm{d}s. \end{array}\right. }\qquad \end{aligned}$$

Substituting (5.16) into expression for $\lambda$, it comes

5.17 $$\begin{aligned} \left( 1- {\mathcal {R}}_{\nu } [\varvec{{\widetilde{\varphi }}}] + \frac{{\mathcal {R}}_\nu [\widetilde{\varvec{\varphi }}]\,{\widetilde{\lambda }}}{\nu +\mu _h+{\widetilde{\lambda }}} \right) \lambda =\frac{ {\mathcal {R}}_\nu [\widehat{\varvec{\varphi }}]\,{\widetilde{\lambda }}}{\nu +\mu _h+{\widetilde{\lambda }}} + \sum _{i\in {\mathcal {I}}} \; \int _0^{\infty } \left\langle \varvec{\beta }_i(\tau ), \widehat{{{\textbf {y}}}}_i(\tau ) \right\rangle \textrm{d}\tau ,\nonumber \\ \end{aligned}$$

where $\displaystyle {\mathcal {R}}_\nu [\varvec{{\widetilde{\varphi }}}]=\sum _{i\in {\mathcal {I}}} {\widetilde{S}}_i\; \int _0^{\infty } \left\langle \varvec{\beta }_i(\tau ), \varvec{\Pi }_i(\tau ,0){{\textbf {q}}}_i \right\rangle \; e^{-\nu \tau }\;\textrm{d}\tau$ and $\widehat{{{\textbf {y}}}}_i(\tau )= e^{-\nu \tau }\varvec{\Pi }_i(\tau ,0)\widehat{{{\textbf {u}}}}_i+ \int _{0}^{\tau } \varvec{\Pi }_i(\tau ,s)\widehat{{{\textbf {I}}}}_i(s) e^{-\nu (\tau -s)}\textrm{d}s$. Therefore, we can isolate $\lambda$ in system (5.17) if and only if $\displaystyle \Delta (\nu ,\varvec{{\widetilde{\varphi }}})=1- {\mathcal {R}}_{\nu } [\varvec{{\widetilde{\varphi }}}] + \frac{{\mathcal {R}}_\nu [\widetilde{\varvec{\varphi }}]\,{\widetilde{\lambda }}}{\nu +\mu _h+{\widetilde{\lambda }}}\ne 0$. $\square$

### Uniform persistence

Our next technical material concerns the uniform persistence of Model (2.5) when ${\mathcal {R}}_0>1$ by using the method developed in Theorem 5.2 in Smith and Thieme (2011).

For the invariant sets of uniform persistence, we introduce

$$\begin{aligned} {\mathcal {M}} ={\mathbb {R}}^n\times \{{{\textbf {0}}}_{{\mathbb {R}}^{2n}}\}\times {\mathcal {M}}_I, \quad \text{ and }\quad \partial {\mathcal {M}}= {\mathcal {X}}_{0+} \setminus {\mathcal {M}}, \end{aligned}$$

where

$$\begin{aligned} {\mathcal {M}}_I=\left\{ {{\textbf {I}}}\in L_+^1((0,\infty ),{\mathbb {R}}^{2n}) \,:\, \sum _{i\in {\mathcal {I}}}\int _0^{\infty } \left\langle \varvec{\beta }_i(\tau ),{{\textbf {I}}}_i(\tau ) \right\rangle \textrm{d}\tau >0 \right\} . \end{aligned}$$

For the unique solution $\varvec{\varphi }=({{\textbf {S}}},{{\textbf {0}}}_{{\mathbb {R}}^{2n}},{{\textbf {I}}})$ of system (2.5) associated to the initial condition $\varvec{\varphi }_0=({{\textbf {S}}}_0,{{\textbf {0}}}_{{\mathbb {R}}^{2n}},{{\textbf {I}}}_0(\cdot ))\in {\mathcal {M}}$, we define $\varvec{\Psi }(t,\varvec{\varphi }_0)=({{\textbf {S}}}(t),{{\textbf {0}}}_{{\mathbb {R}}^{2n}},{{\textbf {I}}}(t,\cdot ))$ the semiflow of Model (2.5) passing through $\varvec{\varphi }_{0}$. Next, we first claim that

#### Claim 5.7

The subsets ${\mathcal {M}}$ and $\partial {\mathcal {M}}$ are positively invariant with respect to the semiflow $\varvec{\Psi }(t,\cdot )$ generated by system (2.5). Furthermore, $\lim \nolimits _{t\rightarrow \infty }\varvec{\Psi }(t,\varvec{\varphi }_{0})=({{\textbf {S}}}^0,{{\textbf {0}}}_{{\mathbb {R}}^{2n}},{{\textbf {0}}}_{L_+^1((0,\infty ),{\mathbb {R}}^{2n})})^T$ for each $\varvec{\varphi }_{0}\in \partial {\mathcal {M}}$.

#### Proof of Claim 5.7

Let $\varvec{\varphi }_{0}=({{\textbf {S}}}_{0},{{\textbf {0}}}_{{\mathbb {R}}^{2n}},{{\textbf {I}}}_{0}(\cdot ))^T\in {\mathcal {M}}$ be given and $\varvec{\Psi }(t,\varvec{\varphi }_{0})=({{\textbf {S}}},{{\textbf {0}}}_{{\mathbb {R}}^{2n}},{{\textbf {I}}}(t,\cdot ))^T,$ the orbit passing through $\varvec{\varphi }_{0}$. Since $\varvec{\varphi }_{0}\in {\mathcal {M}}$, then $\lambda (0)>0$. Through a direct calculation, we have

$$\begin{aligned} \partial _t \lambda (t)= & {} \sum _{i\in {\mathcal {I}}} \int _0^{\infty } \left\langle \varvec{\beta }_i(\tau ),\partial _t {{\textbf {I}}}_i(t,\tau ) \right\rangle \textrm{d}\tau ,\\\ge & {} -(\alpha _0+\omega _0+\mu _h)\lambda (t), \end{aligned}$$

where $\alpha _0=\max _{i\in {\mathcal {I}}}\{\sup \alpha _i^T,\sup \alpha _i^U\}$ and $\omega _0=\max \{{\overline{\omega }}_T^U,{\overline{\omega }}_U^T\}$. Thus, one obtains that

$$\begin{aligned} \lambda (t)\ge e^{-(\alpha _0+\omega _0+\mu _h)}\lambda (0)>0, \end{aligned}$$

for $t\ge 0$. This complete the fact that ${\mathcal {M}}$ is positively invariant.

Now, let $\varvec{\varphi }_{0}\in \partial {\mathcal {M}}$. Since for all $i\in {\mathcal {I}}$, $S_i(t)\le S_i^0$ as *t* is large enough, the comparison principle implies that

5.18 $$\begin{aligned} {{\textbf {I}}}_{i}(t,\tau )\le \widetilde{{{\textbf {I}}}}_{i}(t,\tau ),\quad \forall i\in {\mathcal {I}}, \end{aligned}$$

where $\widetilde{{{\textbf {I}}}}_{i}(t,\tau )$ is the solution of the following system

5.19 $$\begin{aligned} {\left\{ \begin{array}{ll} \displaystyle \widetilde{{{\textbf {I}}}}_i(t,\tau =0)={\widetilde{\lambda }}(t)S_i^0\;{{\textbf {q}}}_i,\\ \widetilde{{{\textbf {I}}}}_i(0,\tau )={{\textbf {I}}}_{i0}(\tau ), \\ \displaystyle \left( \partial _t+\partial _{\tau }\right) \widetilde{{{\textbf {I}}}}_i(t,\tau )= (-{\varvec{e}}\varvec{\omega }(\tau ) -\varvec{\alpha }_i(\tau )-\mu _h+\varvec{\omega }(\tau ))\widetilde{{{\textbf {I}}}}_i(t,\tau ). \\ \end{array}\right. } \end{aligned}$$

By the Volterra formulation, we have from (5.19) that

$$\begin{aligned} \widetilde{{{\textbf {I}}}}_i(t,\tau )=\left\{ \begin{array}{ll} \displaystyle \varvec{\Pi }_i(\tau ,\tau -t)\, {{\textbf {I}}}_{i0}(\tau -t), &{} \hbox {if} \quad t\le \tau , \\ \displaystyle \varvec{\Pi }_i(\tau ,0)\, {{\textbf {q}}}_i\,S_i^0\; {\widetilde{\lambda }}(t-\tau ), &{} \hbox {if}\quad t>\tau , \end{array} \right. \end{aligned}$$

where $${\widetilde{\lambda }}(t)$$ satisfies

$$\begin{aligned} {\widetilde{\lambda }}(t)= & {} \sum _{k\in {\mathcal {I}}} S_k^0\int _0^t \left\langle \varvec{\beta }_k(\tau ), \varvec{\Pi }_k(\tau ,0) {{\textbf {q}}}_k \right\rangle {\widetilde{\lambda }}(t-\tau ) \textrm{d}\tau \\ {}{} & {} +\, \sum _{k\in {\mathcal {I}}}\int _t^{\infty } \left\langle \varvec{\beta }_k(\tau ), \varvec{\Pi }_k(\tau ,\tau -t) {{\textbf {I}}}_{k0}(\tau -t) \right\rangle \textrm{d}\tau . \end{aligned}$$

The initial condition with $\displaystyle \sum _{k\in {\mathcal {I}}}\int _0^{\infty } \left\langle \varvec{\beta }_k(\tau ), {{\textbf {I}}}_{k0}(\tau ) \right\rangle \textrm{d}\tau =0$, leads to

$$\begin{aligned} {\widetilde{\lambda }}(t)= \sum _{k\in {\mathcal {I}}} S_k^0\int _0^t \left\langle \varvec{\beta }_k(\tau ), \varvec{\Pi }_k(\tau ,0) {{\textbf {q}}}_k \right\rangle {\widetilde{\lambda }}(t-\tau ) \textrm{d}\tau . \end{aligned}$$

Since ${\widetilde{\lambda }}(0)=0$, we have ${\widetilde{\lambda }}(t)=0$ for all $t\ge 0$, and then $\widetilde{{{\textbf {I}}}}_{i}(t,\cdot )=0$ for all $i\in {\mathcal {I}}$ and $t\ge 0$. The comparison in (5.18) implies that ${{\textbf {I}}}_{i}(t,\cdot )=0$ for all $i\in {\mathcal {I}}$ and $t\ge 0$ and then $\partial {\mathcal {M}}$ is positively invariant under the semiflow $\varvec{\Psi }(t,\cdot )$. In addition, it is clear for the solution remaining in $\partial {\mathcal {M}}$, we have for all $i\in {\mathcal {I}}$, $S_i\rightarrow S_i^0$. Hence, $\lim \nolimits _{t\rightarrow \infty } \varvec{\Psi }(t,\varvec{\varphi }_{0})=({{\textbf {S}}}^0,{{\textbf {0}}}_{{\mathbb {R}}^{2n}},{{\textbf {0}}}_{L_+^1((0,\infty ),{\mathbb {R}}^{2n})})^T$ for each $\varvec{\varphi }_{0}\in \partial {\mathcal {M}}$. This ends the proof of Claim 5.7. $\square$

Finally, we end this technical material section by establishing the uniform persistence of system (2.5).

#### Theorem 5.8

The semiflow $\{\varvec{\Psi }(t,\cdot )\}_{t\ge 0}$ generated by system (2.5) is uniformly persistent in ${\mathcal {M}}$ with respect to $({\mathcal {M}},\partial {\mathcal {M}})$, that is, there exists a constant $\eta >0$ such that for each $\varvec{\varphi }_{0}\in {\mathcal {M}}$,

$$\begin{aligned} \liminf \limits _{t\rightarrow \infty }{{{\textbf {S}}}(t)}\ge \eta , \end{aligned}$$

and

$$\begin{aligned} \liminf \limits _{t\rightarrow \infty }{\Vert {{\textbf {I}}}(t,\cdot )\Vert _{L^1_+}}\ge \eta \quad \text{ whenever }\quad {\mathcal {R}}_0>1. \end{aligned}$$

Furthermore, there exists compact global attractor ${\mathcal {A}}_1$ in ${\mathcal {M}}$ for the semiflow $\{\varvec{\Psi }(t,\cdot )\}_{t\ge 0}$.

#### Proof

In the following, we will prove that $W^S(\{{{\textbf {E}}}^0\})\cap {\mathcal {M}}=\emptyset$, where

$$\begin{aligned} W^S(\{{{\textbf {E}}}^0\})=\{\varvec{\varphi }_0\in {\mathcal {X}}_{0+}\,:\, \lim \limits _{t\rightarrow +\infty }\varvec{\Psi }(t,\varvec{\varphi }_0)={{\textbf {E}}}^0\}. \end{aligned}$$

Since from Claim 5.7 the disease-free equilibrium ${{\textbf {E}}}^0$ is globally asymptotically stable in $\partial {\mathcal {M}}$, we need only to study the behavior of the solution starting in ${\mathcal {M}}$ in some neighborhood of ${{\textbf {E}}}^0$. To this end, it is sufficient to show that there exists $\sigma >0$ satisfying for each $\varvec{\varphi }\in \{{{\textbf {v}}}\in {\mathcal {M}}\,:\, \Vert {{\textbf {E}}}^0-{{\textbf {v}}}\Vert \le \sigma \}$ there exists $t_0\ge 0$ such that $\Vert \varvec{\Psi }(t,\varvec{\varphi }_0)-{{\textbf {E}}}^0\Vert >\sigma$.

By the way of contradiction, suppose that for each integer $n\ge 0$ there exists a $\varvec{\varphi }_{0}^n=({{\textbf {S}}}_{0}^n,{{\textbf {0}}}_{L^1},{{\textbf {I}}}_{0}^n)\in \{{{\textbf {v}}}\in {\mathcal {M}}\,:\, \Vert {{\textbf {E}}}^0-{{\textbf {v}}}\Vert \le \sigma \}$ such that

$$\begin{aligned} \Vert {{\textbf {E}}}^0-\varvec{\Psi }(t,\varvec{\varphi }_{0}^n)\Vert \le \frac{1}{n+1},\quad \forall t\ge 0. \end{aligned}$$

Denote $\varvec{\Psi }(t,\varvec{\varphi }_{0}^n)=({{\textbf {S}}}^n(t),{{\textbf {0}}}_{{\mathbb {R}}^{2n}},{{\textbf {I}}}^n(t,\cdot ))$, then for all $t\ge 0$ we have

5.20 $$\begin{aligned} |{{\textbf {S}}}^n(t)-{{\textbf {S}}}^0|\le \frac{1}{n+1},\quad \forall t\ge 0. \end{aligned}$$

It follows that for all *i*, we have $S_i^n(t)\ge S_i^0-\frac{1}{n+1}$ for all $t\ge 0$. Consider the following system

$$\begin{aligned} {\left\{ \begin{array}{ll} \displaystyle \left( \partial _t+\partial _{\tau }\right) {{\textbf {I}}}^n(t,\tau )=(-\text {diag}(\varvec{\Phi }(\tau ))+ \text {diag}(\varvec{\omega }(\tau )))\;{{\textbf {I}}}^n(t,\tau ), \\ \displaystyle {{\textbf {I}}}^n(t,0)=\lambda ^n(t)\;\text {diag}({{\textbf {S}}}^n(t))\;{{\textbf {q}}},\\ {{\textbf {S}}}^n(0)={{\textbf {S}}}^n_0,\quad {{\textbf {I}}}^n(0,\cdot )={{\textbf {I}}}^n_{0}(\cdot ),\quad ({{\textbf {S}}}^n_0,{{\textbf {I}}}^n_{0})\in {\mathcal {M}}, \\ \end{array}\right. } \end{aligned}$$

where $\displaystyle \lambda ^n(t)= \sum _{i\in {\mathcal {I}}} \int _0^t \left\langle \varvec{\beta }_i(\tau ), {{\textbf {I}}}^n_i(t,\tau ) \right\rangle \textrm{d}\tau$, ${{\textbf {S}}}_0^n=({{\textbf {S}}}_{i0}^n)_{i\in {\mathcal {I}}}$ and ${{\textbf {I}}}_0^n(\cdot )=({{\textbf {I}}}_{i0}^n(\cdot ))_{i\in {\mathcal {I}}}$. By the comparison principle, we have

5.21 $$\begin{aligned} {{\textbf {I}}}^n(t,\cdot )\ge \widetilde{{{\textbf {I}}}}^n(t,\cdot ), \end{aligned}$$

where $\widetilde{{{\textbf {I}}}}^n(t,\cdot )$ is the solution of the following auxiliary system

$$\begin{aligned} {\left\{ \begin{array}{ll} \displaystyle \left( \partial _t+\partial _{\tau }\right) \widetilde{{{\textbf {I}}}}^n(t,\tau )=(-\text {diag}(\varvec{\Phi }(\tau ))+ \text {diag}(\varvec{\omega }(\tau )))\widetilde{{{\textbf {I}}}}^n(t,\tau ), \\ \displaystyle \widetilde{{{\textbf {I}}}}^n(t,0)={\widetilde{\lambda }}^n(t)\,\text {diag}\left( {{\textbf {S}}}^0-\frac{1}{n+1}I\right) \,{{\textbf {q}}},\\ \widetilde{{{\textbf {I}}}}^n(0,\cdot )={{\textbf {I}}}^n_{0}(\cdot ), \\ \end{array}\right. } \end{aligned}$$

which gives for all *i*,

5.22 $$\begin{aligned} {\left\{ \begin{array}{ll} \displaystyle \left( \partial _t+\partial _{\tau }\right) \widetilde{{{\textbf {I}}}}_i^n(t,\tau )=(-\varvec{\Phi }_i(\tau )+ \varvec{\omega }(\tau ))\widetilde{{{\textbf {I}}}}_i^n(t,\tau ), \\ \displaystyle \widetilde{{{\textbf {I}}}}_i^n(t,0)={\widetilde{\lambda }}^n(t)\,\left( {{\textbf {S}}}_i^0-\frac{1}{n+1}\right) \,{{\textbf {q}}}_i,\\ \widetilde{{{\textbf {I}}}}_i^n(0,\cdot )={{\textbf {I}}}^n_{i0}(\cdot ). \\ \end{array}\right. } \end{aligned}$$

For ease of notation, let us rewrite the system (5.22) as the following form:

5.23 $$\begin{aligned} \frac{d\widetilde{{{\textbf {v}}}}_i^n(t)}{dt}=\left( {\widetilde{A}}_i^n+\widetilde{{\mathcal {L}}}_i^n\right) \widetilde{{{\textbf {v}}}}^n_i(t),\quad \forall t\ge 0, \end{aligned}$$

$\widetilde{{{\textbf {v}}}}^n_i(0)\in \overline{D({\widetilde{A}}^n_i)}$, the closure of $D({\widetilde{A}}^n_i)=\left\{ {{\textbf {0}}}_{{\mathbb {R}}^2}\right\} \times W^{1,1}((0,\infty ),{\mathbb {R}}^2)$, where $\widetilde{{{\textbf {v}}}}^n_i(t)=({{\textbf {0}}}_{{\mathbb {R}}^2},\widetilde{{{\textbf {I}}}}^n_i(t,\cdot ))^t$ and the operators ${\widetilde{A}}^n_i$ and $\widetilde{{\mathcal {L}}}^n_i$ are defined as

$$\begin{aligned} {\widetilde{A}}^n_i({{\textbf {0}}}_{{\mathbb {R}}^2},\widetilde{{{\textbf {I}}}}^n_i)=\Big (-\widetilde{{{\textbf {I}}}}^n_i(0)\,,\,-\partial _{\tau } \widetilde{{{\textbf {I}}}}^n_i+ (-\varvec{\Phi }_i(\tau )+ \varvec{\omega }(\tau ))\; \widetilde{{{\textbf {I}}}}^n_i\Big ) \end{aligned}$$

and

$$\begin{aligned} \widetilde{{\mathcal {L}}}^n_i({{\textbf {0}}}_{{\mathbb {R}}^2},\widetilde{{{\textbf {I}}}}^n_i)=\left( {\widetilde{\lambda }}^n(t)\,\left( {{\textbf {S}}}_i^0-\frac{1}{n+1}\right) \,{{\textbf {q}}}_i\,,\,{{\textbf {0}}}_{L^1((0,\infty ),{\mathbb {R}}^2)}\right) . \end{aligned}$$

Similarly to the proof of Lemma 5.6, we can derive the characteristic equation $\Delta (\nu _n)=0$ for system (5.22), where

$$\begin{aligned} \Delta (\nu _n)=1-\sum _{i\in {\mathcal {I}}}\left( {{\textbf {S}}}_i^0-\frac{1}{n+1}\right) \; \int _0^{\infty } \left\langle \varvec{\beta }_i(\tau ), \varvec{\Pi }_i(\tau ,0){{\textbf {q}}}_i \right\rangle \; e^{-\nu _n \tau }\;\textrm{d}\tau . \end{aligned}$$

Since ${\mathcal {R}}_0>1$, there exists $n_0>0$ large enough such that for $n\ge n_0$,

$$\begin{aligned} {\mathcal {R}}_0^n= \sum _{i\in {\mathcal {I}}}\left( {{\textbf {S}}}_i^0-\frac{1}{n+1}\right) \; \int _0^{\infty } \left\langle \varvec{\beta }_i(\tau ), \varvec{\Pi }_i(\tau ,0){{\textbf {q}}}_i \right\rangle \;\textrm{d}\tau >1. \end{aligned}$$

The largest eigenvalue $\nu _n^*$ of system (5.23) satisfies the characteristic equation $\Delta (\nu _n)=0$. Furthermore, ${\mathcal {R}}_0^n>1$ implies the existence of a dominant eigenvalue $\nu _n^*>0$ such that $\Delta (\nu _n^*)=0$. Therefore, $\nu _{n}^*>0$ is a simple dominant eigenvalue of $({\widetilde{A}}^n_i+\widetilde{{\mathcal {L}}}^n_i)$. From Lemma 5.6, we have shown that $\omega _{0,ess}({\widetilde{A}}^n_i+\widetilde{{\mathcal {L}}}^n_i)\le -\mu _h$ and since the semigroup $\left\{ T_{({\widetilde{A}}^n_i+\widetilde{{\mathcal {L}}}^n_i)}(t)\right\} _{t\ge 0}$ is irreducible, it follows from Corollary 4.6.8 in Magal and Ruan (2018) that $\left\{ T_{({\widetilde{A}}^n_i+ \widetilde{{\mathcal {L}}}^n_i)}(t)\right\} _{t\ge 0}$ has asynchronous exponential growth with intrinsic growth constant $\nu _n^*\in {\mathbb {R}}$. Therefore, using Theorem 3.9 in Magal and McCluskey (2013), we have

$$\begin{aligned} T_{({\widetilde{A}}^n_i+\widetilde{{\mathcal {L}}}^n_i)_0}(t)\,{\widetilde{\Pi }}_{\nu _{n}^*}= {\widetilde{\Pi }}_{\nu _{n}^*}\,T_{({\widetilde{A}}^n_i+\widetilde{{\mathcal {L}}}^n_i)_0}(t)=e^{\nu _n^*t}\,{\widetilde{\Pi }}_{\nu _{n}^*},\quad \forall t\ge 0, \end{aligned}$$

and there exist constants $\varepsilon _0>0$ and $\eta _0>0$ such that

$$\begin{aligned} \Vert T_{({\widetilde{A}}^n_i+\widetilde{{\mathcal {L}}}^n_i)_0}(t)\,(I-{\widetilde{\Pi }}_{\nu _{n}^*})\Vert \le \eta _0e^{(\nu _n^*-\varepsilon _0)t}\,\Vert (I-{\widetilde{\Pi }}_{\nu _{n}^*})\Vert , \quad \forall t\ge 0, \end{aligned}$$

where ${\widetilde{\Pi }}_{\nu _{n}^*}$ is the projector on the generalized eigenspace associated with the largest eigenvalue $\nu _n^*>0$. We deduce that

$$\begin{aligned} {\widetilde{\Pi }}_{\nu _{n}^*}\,{\widetilde{v}}_{i}^n(t)=e^{\nu _n^*t}\,{\widetilde{\Pi }}_{\nu _{n}^*}\,{\widetilde{v}}_{i0}^n. \end{aligned}$$

Since $\nu _n^*>0$, it follows that $\lim \limits _{t\rightarrow \infty }\Vert {\widetilde{\Pi }}_{\nu _{n}^*}\,{\widetilde{v}}_{i}^n(t)\Vert _{L^1}=+\infty$. Therefore, $\lim \limits _{t\rightarrow \infty }\Vert \widetilde{{{\textbf {I}}}}_i^n(t,\cdot )\Vert _{L^1}=+\infty$ and from (5.21), we have $\lim \limits _{t\rightarrow \infty }\Vert {{\textbf {I}}}_i^n(t,\cdot )\Vert _{L^1}=+\infty$, which is a contradiction to the boundedness of the solution. Thus, $W^S(\{{{\textbf {E}}}^0\})\cap {\mathcal {M}}=\emptyset$ and we derive from Theorem 4.2 in Hale and Waltman (1989) that the semiflow $\{\varvec{\Psi }(t,\cdot )\}_{t\ge 0}$ is uniform persistent with respect to the pair $({\mathcal {M}},\partial {\mathcal {M}})$. Moreover, by Theorem 3.7 in Magal and Zhao (2005), there exists a compact global attractor ${\mathcal {A}}_1\subset {\mathcal {M}}$ for the semiflow $\{\varvec{\Psi }(t,\cdot )\}_{t\ge 0}$. $\square$

## Proof of Theorem 2.7

The proof of Theorem 2.7 is decomposed into two parts. The first part is devoted to the global stability of the disease-free equilibrium, while the second part is devoted to the global stability of the endemic equilibrium.

### Proof of Theorem 2.7 (i): global stability of the disease-free equilibrium

When the initial condition of System (2.5) satisfies $\varvec{\varphi }_{0}\in \partial {\mathcal {M}}$, i.e., $\sum _{i\in {\mathcal {I}}}\int _0^{\infty } \left\langle \varvec{\beta }_i(\tau ),{{\textbf {I}}}_{i0}(\tau ) \right\rangle \textrm{d}\tau =0$, by Claim 5.7, it comes that the semiflow $\varvec{\Psi }(t,\varvec{\varphi }_{0})$ generated by system (2.5) is such that $\lim \limits _{t\rightarrow \infty }\varvec{\Psi }(t,\varvec{\varphi }_{0})=({{\textbf {S}}}^0,{{\textbf {0}}}_{{\mathbb {R}}^{2n}},{{\textbf {0}}}_{L_+^1((0,\infty ),{\mathbb {R}}^{2n})})$. It then remain to prove the global stability of the disease-free equilibrium when ${\mathcal {R}}_0\le 1$.

#### Theorem 6.1

If ${\mathcal {R}}_0\le 1$ then, the disease-free equilibrium ${{\textbf {E}}}^0=({{\textbf {S}}}^0,\,{{\textbf {0}}}_{L^1((0,\infty )\times {\mathbb {R}},{\mathbb {R}}^{2n})})$ of system (2.5) is globally asymptotically stable.

#### Proof

By Theorem 5.8, we introduce the following well defined Lyapunov functional $V(t)=V_1(t)+V_2(t)$, with

$$\begin{aligned} V_1(t)= & {} \sum _{k\in {\mathcal {I}}}\left( S_k-S_k^0-S_k^0\ln \frac{S_k}{S_k^0}\right) \quad \text{ and } \quad \\ V_2(t)= & {} \displaystyle \sum _{k\in {\mathcal {I}}} \int _{0}^{\infty }\left( c_k^T(\tau )I^T_k(t,\tau )+ c_k^U(\tau )I^U_k(t,\tau )\right) \textrm{d}\tau , \end{aligned}$$

where ${{\textbf {c}}}_k(\tau )=(c_k^T(\tau ),\; c_k^U(\tau ))^T$ is a vector of positive constants such that

6.1 $$\begin{aligned} {{\textbf {c}}}_k(\tau )={\overline{S}}^0\int _{\tau }^{\infty }\ ^t\varvec{\Pi }_k(\sigma ,\tau ) \varvec{\beta }_k(\sigma )\textrm{d}\sigma , \end{aligned}$$

with $\displaystyle {\overline{S}}^0=\sum _{k\in {\mathcal {I}}} S_k^0$ and ${{\textbf {c}}}_k(\tau )\rightarrow 0$ as $\tau \rightarrow \infty$. From (6.1), we have

6.2 $$\begin{aligned} {\left\{ \begin{array}{ll} {\overline{S}}^0\beta _k^T(\tau )+ \partial _{\tau }c_k^T(\tau )-\gamma _k^T(\tau ) c_k^T(\tau ) + \omega ^T_U(\tau )c_k^U(\tau )=0,\\ {\overline{S}}^0\beta _k^U(\tau )+ \partial _{\tau }c_k^U(\tau )-\gamma _k^U(\tau ) c_k^U(\tau )+ \omega _T^U(\tau )c_k^T(\tau )=0, \end{array}\right. } \end{aligned}$$

with

$$\begin{aligned} \gamma _k^T(\tau )=\alpha ^T_k(\tau )+\omega ^T_U(\tau )+\mu _h\quad \text{ and }\quad \gamma _k^U(\tau )=\alpha ^U_k(\tau )+\omega ^U_T(\tau )+\mu _h. \end{aligned}$$

Differentiating $V_1(t)$ and using $\Lambda _k=\mu _h S_k^0$, we have

$$\begin{aligned} \frac{dV_1(t)}{dt}= & {} \sum _{k\in {\mathcal {I}}}\left( 1-\frac{S^0_k}{S_k}\right) \left( \Lambda _k-S_k(t)\lambda (t) -\mu _h S_k(t)\right) \\= & {} \sum _{k\in {\mathcal {I}}} \Lambda _k\left( 2-\frac{S^0_k}{S_k}-\frac{S_k}{S_k^0} \right) + \sum _{k\in {\mathcal {I}}}\left( \lambda (t)S_k^0- \lambda (t)S_k\right) \\= & {} \sum _{k\in {\mathcal {I}}} \Lambda _k\left( 2-\frac{S^0_k}{S_k}-\frac{S_k}{S_k^0} \right) - \sum _{k\in {\mathcal {I}}} \lambda (t)S_k\\{} & {} +\, \sum _{k\in {\mathcal {I}}} {\overline{S}}^0\int _{0}^{\infty } \Big (\beta _k^T(\tau ) I_k^T(t,\tau ) + \beta _k^U(\tau )I_k^U(t,\tau )\Big )\textrm{d}\tau . \end{aligned}$$

Differentiating $V_2(t)$, we have

$$\begin{aligned} \frac{dV_2(t)}{dt}= & {} \displaystyle \sum _{k\in {\mathcal {I}}} \int _{0}^{\infty }\left( c_k^T(\tau ) \frac{\partial }{\partial t}I^T_k(t,\tau )+ c_k^U(\tau ) \frac{\partial }{\partial t}I^U_k(t,\tau )\right) \textrm{d}\tau \\= & {} - \displaystyle \sum _{k\in {\mathcal {I}}} \int _{0}^{\infty }c_k^T(\tau ) \left( \frac{\partial }{\partial \tau }I_k^T(t,\tau )+ \gamma _k^T(\tau )I_k^{T}(t,\tau )- \omega _T^U(\tau )I_k^{U}(t,\tau )\right) \textrm{d}\tau \\{} & {} -\,\displaystyle \sum _{k\in {\mathcal {I}}} \int _{0}^{\infty }c_k^U(\tau ) \left( \frac{\partial }{\partial \tau }I_k^U(t,\tau )+ \gamma _k^U(\tau )I_k^{U}(t,\tau )- \omega ^T_U(\tau )I_k^{T}(t,\tau )\right) \textrm{d}\tau . \end{aligned}$$

By integrating by parts, we have

$$\begin{aligned} \sum _{k\in {\mathcal {I}}} \int _{0}^{\infty }c_k^T(\tau ) \frac{\partial }{\partial \tau }I_k^T(t,\tau )\;\textrm{d}\tau= & {} - \sum _{k\in {\mathcal {I}}} c_k^T(0)I_k^{T}(t,0) - \sum _{k\in {\mathcal {I}}} \int _{0}^{\infty } \partial _{\tau }c_k^T(\tau )I_k^{T}(t,\tau )\;\textrm{d}\tau \end{aligned}$$

and

$$\begin{aligned} \sum _{k\in {\mathcal {I}}} \int _{0}^{\infty }c_k^U(\tau ) \frac{\partial }{\partial \tau }I_k^U(t,\tau )\;\textrm{d}\tau= & {} - \sum _{k\in {\mathcal {I}}} c_k^U(0)I_k^{U}(t,0) - \sum _{k\in {\mathcal {I}}} \int _{0}^{\infty } \partial _{\tau }c_k^U(\tau )I_k^{U}(t,\tau )\;\textrm{d}\tau . \end{aligned}$$

Replacing these expressions in $\frac{dV_2(t)}{dt}$ and using the fact that $I_k^{T}(t,0)=q_k^T\lambda (t)S_k(t)$ and $I_k^{U}(t,0)=q_k^U\lambda (t)S_k(t)$, we have

$$\begin{aligned} \frac{dV_2(t)}{dt}= & {} \sum _{k\in {\mathcal {I}}} \lambda (t)S_k\Big ( c_k^T(0)q_k^{T}+ c_k^U(0)q_k^{U}\Big )\\{} & {} +\, \displaystyle \sum _{k\in {\mathcal {I}}} \int _{0}^{\infty }\Big ( \partial _{\tau }c_k^T(\tau )-\gamma _k^T(\tau ) c_k^T(\tau ) + \omega ^T_U(\tau )c_k^U(\tau )\Big ) I_k^{T}(t,\tau )\textrm{d}\tau \\{} & {} +\, \displaystyle \sum _{k\in {\mathcal {I}}} \int _{0}^{\infty }\Big ( \partial _{\tau }c_k^U(\tau )-\gamma _k^U(\tau ) c_k^U(\tau )+ \omega _T^U(\tau )c_k^T(\tau )\Big )I_k^{U}(t,\tau ) \textrm{d}\tau . \end{aligned}$$

Finally, combining $\frac{dV_1(t)}{dt}$ and $\frac{dV_2(t)}{dt}$, gathering some terms and using (6.2), it follows that

$$\begin{aligned} \frac{dV(t)}{dt}= & {} \sum _{k\in {\mathcal {I}}} \Lambda _k\left( 2-\frac{S^0_k}{S_k}-\frac{S_k}{S_k^0} \right) + \displaystyle \sum _{k\in {\mathcal {I}}} \lambda (t)S_k\Big ( c_k^T(0)q_k^{T}+ c_k^U(0)q_k^{U}-1\Big )\\{} & {} +\, \displaystyle \sum _{k\in {\mathcal {I}}} \int _{0}^{\infty }\Big ({\overline{S}}^0\beta _k^T(\tau )+ \partial _{\tau }c_k^T(\tau )-\gamma _k^T(\tau ) c_k^T(\tau ) + \omega ^T_U(\tau )c_k^U(\tau )\Big ) I_k^{T}(t,\tau )\textrm{d}\tau \\{} & {} + \,\sum _{k\in {\mathcal {I}}} \int _{0}^{\infty }\Big ( {\overline{S}}^0\beta _k^U(\tau )+ \partial _{\tau }c_k^U(\tau )-\gamma _k^U(\tau ) c_k^U(\tau )+ \omega _T^U(\tau )c_k^T(\tau )\Big )I_k^{U}(t,\tau ) \textrm{d}\tau \\= & {} \sum _{k\in {\mathcal {I}}} \Lambda _k\left( 2-\frac{S^0_k}{S_k}-\frac{S_k}{S_k^0} \right) + \displaystyle \sum _{k\in {\mathcal {I}}} \lambda (t)S_k\Big (\left\langle {{\textbf {c}}}_k(0),{{\textbf {q}}}_k \right\rangle -1\Big ). \end{aligned}$$

Using (6.1), we have

$$\begin{aligned} \sum _{k\in {\mathcal {I}}} S_k^0 \left\langle {{\textbf {c}}}_k(0),{{\textbf {q}}}_k \right\rangle= & {} {\overline{S}}^0\sum _{k\in {\mathcal {I}}} S_k^0\int _0^\infty \left\langle \varvec{\beta }_k(\tau ), \varvec{\Pi }_k(\tau ,0){{\textbf {q}}}_k \right\rangle \textrm{d}\tau ={\overline{S}}^0\,{\mathcal {R}}_0\\= & {} \sum _{k\in {\mathcal {I}}} S_k^0\,{\mathcal {R}}_0\le \sum _{k\in {\mathcal {I}}} S_k^0, \quad \text{ since }\quad {\mathcal {R}}_0\le 1. \end{aligned}$$

That implies that for all *k*, $\left\langle {{\textbf {c}}}_k(0),{{\textbf {q}}}_k \right\rangle \le 1$, when ${\mathcal {R}}_0\le 1$. Therefore, we have $\frac{dV(t)}{dt}\le 0$ when ${\mathcal {R}}_0\le 1$. The strict equality holds only if $S_k(t)=S_k^0$ hold simultaneously with either ${\mathcal {R}}_0=1$ or ${{\textbf {I}}}_k(t,0)=0$. It is easy to verify that largest invariant set in $\left\{ \frac{dV}{dt}=0\right\}$ is the singleton $\{{{\textbf {E}}}^0\}$. Thus, all solutions of system (2.5) converge to the disease-free equilibrium ${{\textbf {E}}}^0$. Hence, ${{\textbf {E}}}^0$ is globally asymptotically stable when ${\mathcal {R}}_0\le 1$. $\square$

### Proof of Theorem 2.7 (ii): global stability of the endemic equilibrium

#### Theorem 6.2

Assume ${\mathcal {R}}_0>1$, then the endemic equilibrium ${{\textbf {E}}}^*=({{\textbf {S}}}^*,{{\textbf {I}}}^*)^t$ of system (2.5) is globally asymptotically stable in ${\mathcal {Y}}_+$.

#### Proof

By Theorem 5.8, we introduce the following well defined Lyapunov functional $L(t)=L_1(t)+L_2(t)+L_3(t)$, where

$$\begin{aligned} L_1(t)= & {} \sum _{k\in {\mathcal {I}}}S_k^* h\left( \frac{S_k}{S_k^*} \right) , \\ L_2(t)= & {} \displaystyle \sum _{k\in {\mathcal {I}}} \int _{0}^{\infty }d_k^T(\tau ) I^{T*}_k(\tau ) h \left( \frac{I^T_k(t,\tau )}{I^{T*}_k(\tau )}\right) \textrm{d}\tau \quad \text{ and } \\ L_3(t)= & {} \displaystyle \sum _{k\in {\mathcal {I}}} \int _{0}^{\infty }d_k^U(\tau ) I^{U*}_k(\tau ) h \left( \frac{I^U_k(t,\tau )}{I^{U*}_k(\tau )}\right) \textrm{d}\tau , \end{aligned}$$

with *h* the function defined by $h(z)=z-1-\ln z$ $(z\in {\mathbb {R}}_+)$, and ${{\textbf {d}}}_k(\tau )=(d_k^T(\tau ),\; d_k^U(\tau ))^T$ a vector of positive constants given by

6.3 $$\begin{aligned} {{\textbf {d}}}_k(\tau )={\overline{S}}^*\int _{\tau }^{\infty }\ ^t\varvec{\Pi }_k(\sigma ,\tau ) \varvec{\beta }_k(\sigma )\textrm{d}\sigma , \end{aligned}$$

where ${\overline{S}}^*=\sum _{k\in {\mathcal {I}}}S_k^*$ and ${{\textbf {d}}}_k(\tau )\rightarrow 0$ as $\tau \rightarrow \infty$. From (6.3), we have

6.4 $$\begin{aligned} \partial _{\tau }{{\textbf {d}}}_k(\tau )- \Big (-\ ^t{\varvec{e}}\varvec{\omega }(\tau )- \varvec{\alpha }_k(\tau )-\mu _h+\varvec{\omega }(\tau )\Big ){{\textbf {d}}}_k(\tau )= -{\overline{S}}^*\varvec{\beta }_k(\tau ). \end{aligned}$$

By using the property of function *h*, we find that the function *L*(*t*) is nonnegative with its global minimum point ${{\textbf {E}}}^*$.

*Step 1:* Differentiating $L_1(t)$ along the solution of system (1.5) and using $\Lambda _k=S_k^*\lambda ^*+\mu _h S_k^*$, we obtain

$$\begin{aligned} \frac{dL_1(t)}{dt}= & {} \sum _{k\in {\mathcal {I}}}\left( 1-\frac{S^*_k}{S_k}\right) \left( \Lambda _k-S_k(t)\lambda (t) -\mu _h S_k(t)\right) \\= & {} \sum _{k\in {\mathcal {I}}}\mu _hS_k^*\left( 2-\frac{S^*_k}{S_k}-\frac{S_k}{S_k^*} \right) + \sum _{k\in {\mathcal {I}}} \lambda ^*S_k^*\left( 1 + \frac{\lambda (t)}{\lambda ^*}- \frac{\lambda (t)S_k}{\lambda ^*S_k^*}-\frac{S^*_k}{S_k}\right) \\= & {} \sum _{k\in {\mathcal {I}}} \mu _hS_k^*\left( 2-\frac{S^*_k}{S_k}-\frac{S_k}{S_k^*} \right) + \sum _{k\in {\mathcal {I}}} \lambda ^*S_k^*\left[ h\left( \frac{\lambda (t)}{\lambda ^*}\right) - h\left( \frac{S_k^*}{S_k}\right) - h\left( \frac{\lambda (t)S_k(t)}{\lambda ^*S_k^*}\right) \right] . \end{aligned}$$

*Step 2:* Note that

6.5 $$\begin{aligned} \partial _{\tau } I_k^{T*}(\tau )= & {} -\gamma _k^T(\tau )I_k^{T*}(\tau )+ \omega _T^U(\tau )I_k^{U*}(\tau )\quad \text{ and }\quad \nonumber \\ \partial _{\tau } I_k^{U*}(\tau )= & {} -\gamma _k^U(\tau )I_k^{U*}(\tau )+ \omega ^T_U(\tau )I_k^{T*}(\tau ). \end{aligned}$$

Using (6.5), we have

6.6 $$\begin{aligned} \left( 1-\frac{I_k^{T*}(\tau )}{I_k^T(t,\tau )}\right) \frac{\partial }{\partial \tau }I_k^T(t,\tau )&= I_k^{T*}(\tau )\frac{\partial }{\partial \tau }h\left( \frac{I_k^{T}(t,\tau )}{I_k^{T*}(\tau )}\right) \nonumber \\&\quad -\, \left( 1-\frac{I_k^{T*}(\tau )}{I_k^T(t,\tau )}\right) \left( \gamma _k^T(\tau )-\omega ^U_T(\tau ) \frac{I_k^{U*}(\tau )}{I_k^{T*}(\tau )}\right) I_k^T(t,\tau ). \end{aligned}$$

Differentiating $L_2(t)$ and, using (6.6) and integration by parts, we obtain

$$\begin{aligned} \frac{dL_2(t)}{dt}= & {} - \sum _{k\in {\mathcal {I}}} \int _{0}^{\infty }d_k^T(\tau ) \left( 1-\frac{I_k^{T*}(\tau )}{I_k^T(t,\tau )}\right) \left( \frac{\partial }{\partial \tau }I_k^T(t,\tau ) +\, \gamma _k^T(\tau )I_k^{T}(t,\tau )- \omega _T^U(\tau )I_k^{U}(t,\tau )\right) \textrm{d}\tau \\= & {} -\sum _{k\in {\mathcal {I}}} \int _{0}^{\infty }d_k^T(\tau )\left[ I_k^{T*}(\tau )\frac{\partial }{\partial \tau }h\left( \frac{I_k^{T}(t,\tau )}{I_k^{T*}(\tau )}\right) - \omega _T^U(\tau )I_k^{U*}(\tau ) -\omega _T^U(\tau )I_k^{U}(t,\tau ) \right. \\{} & {} +\,\left. \omega _T^U(\tau ,x)I_k^{U*}(\tau ) \frac{I_k^{T}(t,\tau )}{I_k^{T*}(\tau )}+ \omega _T^U(\tau )I_k^{T*}(\tau ) \frac{I_k^{U}(t,\tau )}{I_k^{T}(t,\tau )}\right] \textrm{d}\tau \\= & {} \left. - \sum _{k\in {\mathcal {I}}} d_k^T(\tau ) I_k^{T*}(\tau )\;h\left( \frac{I_k^{T}(t,\tau )}{I_k^{T*}(\tau )} \right) \right| _{\tau =0}^{\tau =\infty }\\{} & {} +\,\sum _{k\in {\mathcal {I}}} \int _{0}^{\infty }d_k^T(\tau ) \omega _T^U(\tau )I_k^{U*}(\tau ) \left[ 1+ \frac{I_k^{U}(t,\tau )}{I_k^{U*}(\tau )}- \frac{I_k^{T}(t,\tau )}{I_k^{T*}(\tau )}- \frac{I_k^{T*}(\tau )I_k^{U}(t,\tau )}{I_k^{T}(t,\tau )I_k^{U*}(\tau )}\right] \textrm{d}\tau \\{} & {} +\, \sum _{k\in {\mathcal {I}}} \int _{0}^{\infty } h\left( \frac{I_k^{T}(t,\tau )}{I_k^{T*}(\tau )}\right) \Big (I_k^{T*}(\tau )\partial _{\tau }d_k^T(\tau )+ d_k^T(\tau ) \partial _{\tau }I_k^{T*}(\tau )\Big )\textrm{d}\tau \\= & {} \sum _{k\in {\mathcal {I}}} d_k^T(0) I_k^{T*}(0)\;h\left( \frac{I_k^{T}(t,0)}{I_k^{T*}(0)}\right) \\{} & {} +\, \sum _{k\in {\mathcal {I}}} \int _{0}^{\infty }d_k^T(\tau ) \omega _T^U(\tau )I_k^{U*}(\tau ) \left[ 1+ \frac{I_k^{U}(t,\tau )}{I_k^{U*}(\tau )}- \frac{I_k^{T}(t,\tau )}{I_k^{T*}(\tau )}- \frac{I_k^{T*}(\tau )I_k^{U}(t,\tau )}{I_k^{T}(t,\tau )I_k^{U*}(\tau )}\right] \textrm{d}\tau \\{} & {} +\,\sum _{k\in {\mathcal {I}}} \int _{0}^{\infty } h\left( \frac{I_k^{T}(t,\tau )}{I_k^{T*}(\tau )}\right) \Big (I_k^{T*}(\tau )\partial _{\tau }d_k^T(\tau )+ d_k^T(\tau ) \partial _{\tau }I_k^{T*}(\tau )\Big )\textrm{d}\tau . \end{aligned}$$

Since $I_k^{T*}(0)=q_k^T\lambda ^*S_k^*$ and $I_k^{T}(t,0)=q_k^T\lambda (t)S_k(t)$, then we have

$$\begin{aligned} \frac{dL_2(t)}{dt}= & {} \sum _{k\in {\mathcal {I}}} \lambda ^*S_k^*\; d_k^T(0) q_k^T\; h\left( \frac{\lambda (t)S_k(t)}{\lambda ^*S_k^*} \right) \\{} & {} + \sum _{k\in {\mathcal {I}}} \int _{0}^{\infty }d_k^T(\tau ) \omega _T^U(\tau )I_k^{U*}(\tau ) \left[ 1+ \frac{I_k^{U}(t,\tau )}{I_k^{U*}(\tau )}- \frac{I_k^{T}(t,\tau )}{I_k^{T*}(\tau )}- \frac{I_k^{T*}(\tau )I_k^{U}(t,\tau )}{I_k^{T}(t,\tau )I_k^{U*}(\tau )}\right] \textrm{d}\tau \\{} & {} +\int _{{\mathbb {R}}}\sum _{k\in {\mathcal {I}}} \int _{0}^{\infty } h\left( \frac{I_k^{T}(t,\tau )}{I_k^{T*}(\tau )}\right) \Big (I_k^{T*}(\tau )\partial _{\tau }d_k^T(\tau )+ d_k^T(\tau ) \partial _{\tau }I_k^{T*}(\tau )\Big )\textrm{d}\tau . \end{aligned}$$

By a similarly manner, the derivative of $L_3(t)$ gives

$$\begin{aligned} \frac{dL_3(t)}{dt}= & {} \sum _{k\in {\mathcal {I}}} \lambda ^*S_k^*\; d_k^U(0) q_k^U\; h\left( \frac{\lambda (t)S_k(t)}{\lambda ^*S_k^*} \right) \\{} & {} +\, \sum _{k\in {\mathcal {I}}} \int _{0}^{\infty }d_k^U(\tau ) \omega ^T_U(\tau )I_k^{T*}(\tau ) \left[ 1+ \frac{I_k^{T}(t,\tau )}{I_k^{T*}(\tau )}- \frac{I_k^{U}(t,\tau )}{I_k^{U*}(\tau )}- \frac{I_k^{U*}(\tau )I_k^{T}(t,\tau )}{I_k^{U}(t,\tau )I_k^{T*}(\tau )}\right] \textrm{d}\tau \\{} & {} +\,\sum _{k\in {\mathcal {I}}} \int _{0}^{\infty } h\left( \frac{I_k^{U}(t,\tau )}{I_k^{U*}(\tau )}\right) \Big (I_k^{U*}(\tau )\partial _{\tau }d_k^U(\tau )+ d_k^U(\tau ) \partial _{\tau }I_k^{U*}(\tau )\Big )\textrm{d}\tau . \end{aligned}$$

*Step 3:* Finally, combining $\frac{dL_1(t)}{dt}$, $\frac{dL_2(t)}{dt}$ and $\frac{dL_3(t)}{dt}$, we obtain

$$\begin{aligned} \frac{dL(t)}{dt}= & {} \sum _{k\in {\mathcal {I}}} \mu _hS_k^*\left( 2-\frac{S^*_k}{S_k}-\frac{S_k}{S_k^*} \right) + \sum _{k\in {\mathcal {I}}} \lambda ^*S_k^*\left[ h\left( \frac{\lambda (t)}{\lambda ^*}\right) - h\left( \frac{S_k^*}{S_k}\right) - h\left( \frac{\lambda (t)S_k(t)}{\lambda ^*S_k^*}\right) \right] \\{} & {} +\,\sum _{k\in {\mathcal {I}}} \lambda ^*S_k^* \left\langle {{\textbf {d}}}_k(0),{{\textbf {q}}}_k \right\rangle h\left( \frac{\lambda (t)S_k(t)}{\lambda ^*S_k^*} \right) \\{} & {} +\,\sum _{k\in {\mathcal {I}}} \int _{0}^{\infty } h\left( \frac{I_k^{T}(t,\tau )}{I_k^{T*}(\tau )}\right) \Big (I_k^{T*}(\tau )\partial _{\tau }d_k^T(\tau )+ d_k^T(\tau ) \partial _{\tau }I_k^{T*}(\tau )\Big )\textrm{d}\tau \\{} & {} +\,\sum _{k\in {\mathcal {I}}} \int _{0}^{\infty } h\left( \frac{I_k^{U}(t,\tau )}{I_k^{U*}(\tau )}\right) \Big (I_k^{U*}(\tau )\partial _{\tau }d_k^U(\tau )+ d_k^U(\tau ) \partial _{\tau }I_k^{U*}(\tau )\Big )\textrm{d}\tau \\{} & {} + \,\sum _{k\in {\mathcal {I}}} \int _{0}^{\infty }d_k^T(\tau ) \omega _T^U(\tau )I_k^{U*}(\tau ) \left[ 1+ \frac{I_k^{U}(t,\tau )}{I_k^{U*}(\tau )}- \frac{I_k^{T}(t,\tau )}{I_k^{T*}(\tau )}- \frac{I_k^{T*}(\tau )I_k^{U}(t,\tau )}{I_k^{T}(t,\tau )I_k^{U*}(\tau )}\right] \textrm{d}\tau \\{} & {} +\, \sum _{k\in {\mathcal {I}}} \int _{0}^{\infty }d_k^U(\tau ) \omega ^T_U(\tau )I_k^{T*}(\tau ) \left[ 1+ \frac{I_k^{T}(t,\tau )}{I_k^{T*}(\tau )}- \frac{I_k^{U}(t,\tau )}{I_k^{U*}(\tau )}- \frac{I_k^{U*}(\tau )I_k^{T}(t,\tau )}{I_k^{U}(t,\tau )I_k^{T*}(\tau )}\right] \textrm{d}\tau . \end{aligned}$$

We observe that $d_k^T(\tau )$ and $d_k^U(\tau )$ satisfy

6.7 $$\begin{aligned} d_k^U(\tau )\omega _U^T(\tau )I_k^{T*}(\tau )=d_k^T(\tau ) \omega _T^U(\tau )I_k^{U*}(\tau ). \end{aligned}$$

By using (6.7), (6.5), and (6.4), we obtain

6.8 $$\begin{aligned} \partial _{\tau }d_k^{\vartheta }(\tau )I_k^{\vartheta *}(\tau )+ d_k^{\vartheta }(\tau ) \partial _{\tau }I_k^{\vartheta *}(\tau )=-{\overline{S}}^*\beta ^{\vartheta }_k(\tau ) I_k^{\vartheta *}(\tau ), \quad \vartheta =\{T,U\}. \end{aligned}$$

Moreover, by using (5.7) and (5.8), we have

6.9 $$\begin{aligned} \lambda ^*=\lambda ^*\sum _{k\in {\mathcal {I}}} S_k^*\int _0^\infty \left\langle \varvec{\beta }_k(\tau ), \varvec{\Pi }_k(\tau ,0){{\textbf {q}}}_k\right\rangle \textrm{d}\tau . \end{aligned}$$

Thus, using (6.3) and (6.9), it can be verified that

6.10 $$\begin{aligned} \sum _{k\in {\mathcal {I}}} \lambda ^*S_k^* \left\langle {{\textbf {d}}}_k(0),{{\textbf {q}}}_k \right\rangle= & {} \lambda ^*{\overline{S}}^*\sum _{k\in {\mathcal {I}}} S_k^*\int _0^\infty \left\langle \varvec{\beta }_k(\tau ), \varvec{\Pi }_k(\tau ,0){{\textbf {q}}}_k \right\rangle \textrm{d}\tau \nonumber \\= & {} \lambda ^*{\overline{S}}^*=\sum _{k\in {\mathcal {I}}}\lambda ^* S_k^*, \end{aligned}$$

which implies that for all *k*, $\left\langle {{\textbf {d}}}_k(0),{{\textbf {q}}}_k \right\rangle =d_k^T(0) q_k^T+ d_k^U(0) q_k^U= 1$. In addition, note that

6.11 $$\begin{aligned} \sum _{k\in {\mathcal {I}}}\lambda ^*S_k^*\; h\left( \frac{\lambda (t)}{\lambda ^*}\right)= & {} \sum _{k\in {\mathcal {I}}} \int _{0}^{\infty }{\overline{S}}^* \Big (\beta _k^T(\tau )I_k^{T*}(\tau )\nonumber \\{} & {} +\, \beta _k^U(\tau )\,I_k^{U*}(\tau )\Big )\;h\left( \frac{\lambda (t)}{\lambda ^*}\right) \textrm{d}\tau . \end{aligned}$$

Replacing (6.7), (6.8), (6.10) and (6.11) in $\frac{dL(t)}{dt}$ and gathering some terms, we obtain

$$\begin{aligned} \frac{dL(t)}{dt}= & {} \sum _{k\in {\mathcal {I}}} \mu _hS_k^*\left( 2-\frac{S^*_k}{S_k}-\frac{S_k}{S_k^*} \right) - \sum _{k\in {\mathcal {I}}} \lambda ^*S_k^*\; h\left( \frac{S_k^*}{S_k}\right) \\{} & {} +\,\sum _{k\in {\mathcal {I}}} \int _{0}^{\infty } {\overline{S}}^*\beta _k^T(\tau )\,I_k^{T*}(\tau )\left[ h\left( \frac{\lambda (t)}{\lambda ^*}\right) -h\left( \frac{I_k^{T}(t,\tau )}{I_k^{T*}(\tau )}\right) \right] \textrm{d}\tau \\{} & {} +\,\sum _{k\in {\mathcal {I}}} \int _{0}^{\infty } {\overline{S}}^*\beta _k^U(\tau )\,I_k^{U*}(\tau ) \left[ h\left( \frac{\lambda (t)}{\lambda ^*}\right) -h\left( \frac{I_k^{U}(t,\tau )}{I_k^{U*}(\tau )}\right) \right] \textrm{d}\tau \\{} & {} +\, \displaystyle \sum _{k\in {\mathcal {I}}} \int _{0}^{\infty } d_k^T(\tau )\omega ^U_T(\tau )I_k^{U*}(\tau ) \left( 2- \frac{I_k^{T*}(\tau )}{I_k^{T}(t,\tau )} \frac{I_k^{U}(t,\tau )}{I_k^{U*}(\tau )}- \frac{I_k^{U*}(\tau )}{I_k^{U}(t,\tau )} \frac{I_k^{T}(t,\tau )}{I_k^{T*}(\tau )}\right) \textrm{d}\tau . \end{aligned}$$

Note that

$$\begin{aligned} h\left( \frac{\lambda (t)}{\lambda ^*}\right) -h\left( \frac{I_k^{\vartheta }(t,\tau )}{I_k^{\vartheta *}(\tau )}\right)= & {} - h\left( \frac{I_k^{\vartheta }(t,\tau ) \lambda ^*}{I_k^{\vartheta *}(\tau )\lambda (t)}\right) \\{} & {} +\, \left( \frac{\lambda (t)}{\lambda ^*}-1\right) \left( 1-\frac{I_k^{\vartheta }(t,\tau ) \lambda ^*}{I_k^{\vartheta *}(\tau )\lambda (t)}\right) ,\quad \vartheta =\{T,U\}. \end{aligned}$$

Hence, we have

$$\begin{aligned} \frac{dL(t)}{dt}= & {} \sum _{k\in {\mathcal {I}}} \mu _hS_k^*\left( 2-\frac{S^*_k}{S_k}-\frac{S_k}{S_k^*} \right) -\sum _{k\in {\mathcal {I}}} \int _{0}^{\infty } {\overline{S}}^*\beta _k^T(\tau )\,I_k^{T*}(\tau )\; h\left( \frac{I_k^{T}(t,\tau ) \lambda ^*}{I_k^{T*}(\tau )\lambda (t)}\right) \textrm{d}\tau \\{} & {} -\,\sum _{k\in {\mathcal {I}}} \lambda ^*S_k^*\; h\left( \frac{S_k^*}{S_k}\right) -\sum _{k\in {\mathcal {I}}} \int _{0}^{\infty } {\overline{S}}^*\beta _k^U(\tau )\,I_k^{U*}(\tau )\; h\left( \frac{I_k^{U}(t,\tau ) \lambda ^*}{I_k^{U*}(\tau )\lambda (t)}\right) \textrm{d}\tau \\{} & {} +\,\sum _{k\in {\mathcal {I}}} \int _{0}^{\infty } d_k^T(\tau )\omega ^U_T(\tau )I_k^{U*}(\tau ) \left( 2- \frac{I_k^{T*}(\tau )}{I_k^{T}(t,\tau )} \frac{I_k^{U}(t,\tau )}{I_k^{U*}(\tau )}- \frac{I_k^{U*}(\tau )}{I_k^{U}(t,\tau )} \frac{I_k^{T}(t,\tau )}{I_k^{T*}(\tau )}\right) \textrm{d}\tau \\{} & {} +\,\left( \frac{\lambda (t)}{\lambda ^*}-1\right) \sum _{k\in {\mathcal {I}}}\int _{0}^{\infty }\left[ {\overline{S}}^*\beta _k^T(\tau )I_k^{T*}(\tau ) \left( 1- \frac{I_k^{T}(t,\tau )\, \lambda ^*}{I_k^{T*}(\tau )\,\lambda (t)}\right) \right. \\{} & {} + \,\left. {\overline{S}}^*\beta _k^U(\tau ,)I_k^{U*}(\tau ) \left( 1- \frac{I_k^{U}(t,\tau )\, \lambda ^*}{I_k^{U*}(\tau )\,\lambda (t)}\right) \right] \textrm{d}\tau . \end{aligned}$$

Note that

$$\begin{aligned}{} & {} \sum _{k\in {\mathcal {I}}}\int _{0}^{\infty }\left[ {\overline{S}}^*\beta _k^T(\tau )I_k^{T*}(\tau ) \left( 1- \frac{I_k^{T}(t,\tau )\, \lambda ^*}{I_k^{T*}(\tau )\,\lambda (t)}\right) \right. \\{} & {} \left. \quad + {\overline{S}}^*\beta _k^U(\tau )I_k^{U*}(\tau ) \left( 1- \frac{I_k^{U}(t,\tau )\, \lambda ^*}{I_k^{U*}(\tau )\,\lambda (t)}\right) \right] \textrm{d}\tau =0. \end{aligned}$$

Finally, we have

$$\begin{aligned} \frac{dL(t)}{dt}= & {} \sum _{k\in {\mathcal {I}}} \mu _hS_k^*\left( 2-\frac{S^*_k}{S_k}-\frac{S_k}{S_k^*} \right) -\sum _{k\in {\mathcal {I}}} \int _{0}^{\infty } {\overline{S}}^*\beta _k^T(\tau )\,I_k^{T*}(\tau )\; h\left( \frac{I_k^{T}(t,\tau ) \lambda ^*}{I_k^{T*}(\tau )\lambda (t)}\right) \textrm{d}\tau \\{} & {} -\, \sum _{k\in {\mathcal {I}}} \lambda ^*S_k^*\; h\left( \frac{S_k^*}{S_k}\right) -\sum _{k\in {\mathcal {I}}} \int _{0}^{\infty } {\overline{S}}^*\beta _k^U(\tau )\,I_k^{U*}(\tau )\; h\left( \frac{I_k^{U}(t,\tau ) \lambda ^*}{I_k^{U*}(\tau )\lambda (t)}\right) \textrm{d}\tau \\{} & {} +\,\sum _{k\in {\mathcal {I}}} \int _{0}^{\infty } d_k^T(\tau )\omega ^U_T(\tau )I_k^{U*}(\tau ) \left( 2- \frac{I_k^{T*}(\tau )}{I_k^{T}(t,\tau )} \frac{I_k^{U}(t,\tau )}{I_k^{U*}(\tau )}- \frac{I_k^{U*}(\tau )}{I_k^{U}(t,\tau )} \frac{I_k^{T}(t,\tau )}{I_k^{T*}(\tau )}\right) \textrm{d}\tau . \end{aligned}$$

Thus, $\dfrac{dL(t)}{dt}\le 0$ with equality if and only if $S_k(t)=S_k^*$, $I_k^{T}(t,\tau )=I_k^{T*}(\tau )$ and $I_k^{U}(t,\tau )=I_k^{U*}(\tau )$. Then, it can be verified that largest invariant set in $\left\{ \frac{dL}{dt}=0\right\}$ is the singleton $\{{{\textbf {E}}}^*\}$. It follows that the compact global attractor ${\mathcal {A}}_0$, stated by Lemma 5.4, is such that ${\mathcal {A}}_0=\{{{\textbf {E}}}^*\}$. Therefore, the endemic equilibrium ${{\textbf {E}}}^*$ is globally asymptotically stable in ${\mathcal {Y}}_+$ when ${\mathcal {R}}_0> 1$. $\square$
